# R-Gada: a fast and flexible pipeline for copy number analysis in association studies

**DOI:** 10.1186/1471-2105-11-380

**Published:** 2010-07-16

**Authors:** Roger Pique-Regi, Alejandro Cáceres, Juan R González

**Affiliations:** 1 Signal and Image Processing Institute, Viterbi School of Engineering, University of Southern California, EEB 400, 3740 McClintock Ave, Los Angeles, CA 90089-2564, USA; 2 Division of Hematology - Oncology, Department of Pediatrics, Childrens Hospital Los Angeles, 4650 Sunset Boulevard Los Angeles, CA 90027, USA; 3 Center for Research in Environmental Epidemiology (CREAL), Doctor Aiguader, 88, Barcelona, 08003, Spain; 4 Institut Municipal d'Investigació Mèdica (IMIM), Doctor Aiguader, 88, Barcelona, 08003, Spain; 5 CIBER Epidemiología y Salud Pública (CIBERESP), Doctor Aiguader, 88, Barcelona, 08003, Spain

## Abstract

**Background:**

Genome-wide association studies (GWAS) using Copy Number Variation (CNV) are becoming a central focus of genetic research. CNVs have successfully provided target genome regions for some disease conditions where simple genetic variation (i.e., SNPs) has previously failed to provide a clear association.

**Results:**

Here we present a new R package, that integrates: (i) data import from most common formats of Affymetrix, Illumina and aCGH arrays; (ii) a fast and accurate segmentation algorithm to call CNVs based on Genome Alteration Detection Analysis (GADA); and (iii) functions for displaying and exporting the Copy Number calls, identification of recurrent CNVs, multivariate analysis of population structure, and tools for performing association studies. Using a large dataset containing 270 HapMap individuals (Affymetrix Human SNP Array 6.0 Sample Dataset) we demonstrate a flexible pipeline implemented with the package. It requires less than one minute per sample (3 million probe arrays) on a single core computer, and provides a flexible parallelization for very large datasets. Case-control data were generated from the HapMap dataset to demonstrate a GWAS analysis.

**Conclusions:**

The package provides the tools for creating a complete integrated pipeline from data normalization to statistical association. It can effciently handle a massive volume of data consisting of millions of genetic markers and hundreds or thousands of samples with very accurate results.

## Background

High resolution oligonucleotide array platforms with millions of markers have enabled the study of copy number variation (CNV). CNVs are alterations of the genome in which small segments of DNA sequence are duplicated (gained) or deleted (lost) [1-5]. These alterations can affect regulatory regions or coding portions of a gene, and have been found associated with a number of genetic disorders and some complex heritable diseases [6].

In contrast to SNPs, which rely on having linkage disequilibrium with the underlying causal mutation, CNVs are more likely to point the underlying biological cause that affects the phenotype of interest. This is because the duplication or deletion can readily explain a gain or loss in gene expression levels. While it has been shown that that common CNVs can be tagged well with SNPs (77%) [4,5], Conrad et al. [5] also argue for the need to consider all classes of variation (SNPs and all structural variants, common and rare) in genome wide association studies. In this context, CNVs affecting small regions (in the order of a few kilobases) or structurally complex CNVs (e.g., CNVs without shared boundaries, or nested CNVs) as seen by [7] require high resolution microarrays (millions of probes) and very accurate techniques in placing the copy number change locations. Detection of rare CNVs also requires large sample sizes making essential computationally efficient tools for the CNV extraction.

The complete analysis of CNV association requires three main steps: i) normalization, ii) segmentation, and iii) common alteration and association analysis. The objective of the normalization is to clean as much as possible the data from all known sources of experimental variation unrelated to copy number changes. In contrast to SNP association studies, where we can perform genotyping for each probe individually after normalization, studies using CNVs require the identification of contiguous stretches of probes with the same underlying copy number change. Thus, CNV association studies require a more complex pipeline. In this paper we present a new R package (R-GADA) that facilitates the implementation of a complete pipeline from data normalization to the final CNV association analysis. Since data normalization is highly specific of each microarray technology the package can import normalized data from several packages such as aroma. affymetrix and tools provided by Illumina and Affymetrix. The segmentation algorithm implemented in the package is based on the Genome alteration detection analysis (GADA) [8]. Compared with circular binary segmentation (CBS) [9], one of the most accurate segmentation algorithms available, GADA has similar or better accuracy and is several orders of magnitude faster. Recently, GADA has been used by [5] and [10] to analyze very large data sets. GADA had a limited availability as a C/Matlab library, and lacked a complete pipeline to facilitate the analysis that the presented package provides. The package presents new functionality for automatically splitting the data in files for each sample and chromosome, which can then be analyzed in parallel in a multicore computer. After fitting the GADA model, R-GADA offers efficient methods for adjusting the final segmentation sensitivity and false positive rate, as well as a complete set of tools for visualizing and reporting copy number alterations. Most prominently, R-GADA offers tools for identifying population structure in the CNV data that can be taken into account on the final case-control CNV association study. The pipeline offers a flexible computational framework that can easily accommodate changes in any stage of the analysis to fulfill the requirements of the study.

**Figure 1:**
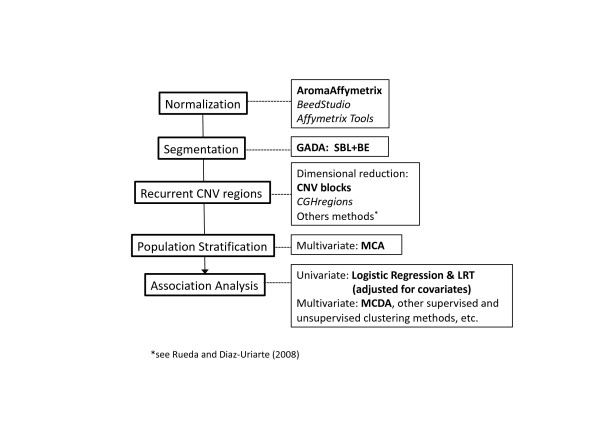
**Pipeline analysis**. Analysis strategies for CNV association studies. On the left, we show the main steps of the pipeline, and on right the algorithms used in each step. The algorithms in bold are explicitly used in the present paper; alternatives procedures, easily adaptable in the pipeline, are also shown.

The package is demonstrated on a large dataset consisting of 270 Hapmap samples (Affymetrix Human SNP 6.0 array sample data). We generated case-control data associated with some CNVs illustrating a potential scenario encountered on GWAS using CNVs. The latter exemplifies the entire analysis process from data normalization, segmentation, recurrent CNV analysis, population structure correction, to the final statistical association analysis; see the illustration of pipeline analysis in Figure 1. More examples are provided through the R-GADA user manual and the Google group http://groups.google.com/group/gadaproject used for providing support.

## Software main features

### Importing normalized data to gada

Data can be imported to gada from Illumina, Affymetrix or any other platform that provides information about ratio intensities such as aCGH. We have implemented three different functions for this purpose: setupGADAIllumina. setupGADAaffy and setupGADAgeneral. setupGADAIllumina function uses information obtained from BeadStudio tools http://www.illumina.com. The software allows information to be provided in either a unique text file or a file per individual (or groups of individuals). Alternatively, setupGADAaffy can import data from the Affymetrix Genotyping Console 3.0 (GTC3) http://www.affymetrix.com that extracts normalized *log*_2 _ratio intensities from a collection of CEL files. The gada package also handles files containing *log*_2 _ratios of other platforms, such as aCGH with the function setupGADAgeneral, which can also be used for cases without chromosome position information. Throughout this paper we interchangeably use the terms *log*_2 _ratios or hybridization intensity ratios when we refer to these normalized values.

### Breakpoint identification using sparse Bayesian learning (SBL)

**Figure 2:**
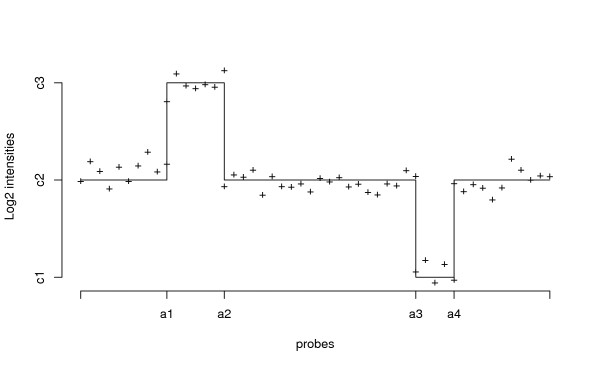
**Probe *log*_2 _ratio intensities**. Schematic representation of probe *log*_2 _ratio intensities with two underlying CNVs. Four breakpoints (*a*1*,a*2*,a*3 and *a*4) separate two altered segments with average intensities *c*3 and *c*1, from the unaltered segments with baseline *c*2.

The underlying model for the DNA copy number and the hybridization ratio intensities observed for each probe in the array is illustrated in Figure 2. The number of copies for each autosomal portion of the human genome is generally 2 but, sometimes, small portions of the DNA are duplicated or deleted originating CNV polymorphisms.

The *log*_2 _ratio of the array probes increases with the number of times its underlying DNA sequence is present in the genome. The normalization step, which is specific of each array platform, should correct for inherent array biases; so contiguous probes sampling the same CNV have the same *log*_2 _ratio average.

Thus, the problem of copy number detection can be casted as a segmentation analysis, where the objective is to identify the set of breakpoints and copy number values that most likely originated the observed data. In the GADA model, the experimental data *y *is explained by a copy number signal *x*, made of piece wise constant segments, and a random error ∈,

(1) y=x+ϵ=Fw+ϵ

The piece-wise component can be conveniently decomposed on a special base F = {*f*_*m*_}_*m *= 0.. *M*-1 _of step functions at probe *m*. If *x *has very few breakpoints, then most of the coefficients of the expansion will be zero, and thus the vector of weights **w **will be sparse.

Using sparse Bayesian learning (SBL) [11], this sparse vector of weights can be computed as a maximum a posteriori estimate

(2) $$\overset{\wedge }{w}=\arg\underset{{}_{w}}{\min}-\log p(y|w)-\log p(w|\alpha )-\log p(\alpha ),$$

where the first two terms are normally distributed - *N*(***Fw***, *σ1*) and $\Pi_{m}N(w_{m}|0,\alpha_{m}^{-1})$ respectively -and the vector of hyperparameters α follows a gamma distribution Π*_m_*Γ (*α_m_*|*a,b*). While α and other distribution parameters are estimated from the data, the parameters *a *and *b *are directly controlled by the user.

Typically *b *is set to zero as an uninformative prior, and *a *takes values within the range 0.2 and 0.8. The user then adjusts the level of sparseness solely with the parameter *a*. The reader is referred to Pique-Regi et al. [8] for more details on the application of Sparse Bayesian Learning (SBL) to segmentation analysis.

### Backward elimination (BE)

The significance of each segment's breakpoint is assessed with the *t*-statistic [8], computed with the parameter estimates provided by the SBL step.

(3) $$t_{j}=\frac{\left|\overset{\wedge }{w}j\right|}{\sqrt{\sigma^{2}{\left[F_{I}F_{I}\right]}^{-1}}}.$$

where *F_I _*is the matrix representation of the reduced basis *F *for which *w_j _≠ *0. Breakpoints with small *t *are discarded with a backward elimination (BE) procedure, until the segment with lowest *t *achieves a predefined threshold *T*. This backward elimination ranking of breakpoints with the adjustment of T is obtained with a very small computational cost.

The SBL and BE procedures make no assumptions on the amplitude of the reconstructed segments. The objective is to provide a nearly optimal set of amplitudes and breakpoint positions that best fits the *log*_2 _ratios observed in the array. Once the breakpoints are fixed, in order to achieve the minimal residual error, the amplitude corresponding to each segment is given by the average hybridization level of all the probes that fall inside that segment. The consequence of this model, is that segments that correspond to the same underlying copy number state may be given a different reconstruction amplitude; and, an additional step has to be used to classify these segments into a copy number (0, 1, 2, 3, 4, ...) or alteration status (*Non-Altered*, *Gain *and *Loss*). Here, we adopt the latter three state classification strategy and focus on alterations not comprising the extent of a whole chromosome. Hence, we first estimate the base-line *Non-Altered *amplitude by calculating the median value of all reconstructed segment amplitudes in a chromosome. Then, we use the same threshold T to classify all the segments into *Gain *(*Loss*) state if the segment amplitude is significantly above (below) the base-line amplitude, or into Non-altered state otherwise. This approach classifies the relative copy number state of each segment with respect the number of copies the chromosome has. For cases, where whole chromosome alterations are expected, the base-line amplitude can be fixed by the user.

### Multivariate analysis

The copy number calls generated in the previous section are categorical variables with three levels: loss, no-change and gain in copy number. For this data, Multiple Correspondence Analysis (MCA) [12] can be used as a *unsupervised *group classification. One of features of MCA is its principle of distributional equivalence, which assures invariance in the results when rows or columns with identical conditional proportions are merged. This allows the identification of common CNV regions that can be used in the analysis without loss of information. Note that this feature is not required in SNP analysis that use PCA. A *supervised *discrimination of CNV maps can also be performed with a Multiple Correspondence Discriminant Analysis (MCDA). In this case, CNVs that are important for the discrimination can be ranked according to their correlation to the class direction (λ) on the *k*-th principal axis subspace [13]

(4) $$\rho_{j}(\lambda )=\frac{\sum_{i=1}^{l}V_{i}(\lambda )(x_{ij}-x_{j})}{\sqrt{\sum_{i=1}^{l}V_{i}{\left(\lambda \right)}^{2}\sum_{i=1}^{l}{\left(x_{ij}-x_{j}\right)}^{2}}},$$

rather than to the axes themselves. In this equation *V_i_*(*k*) is the coordinate of class centroid *i *in the direction of class λ; *x_ij _*is the aggregated indicator matrix of segment callings for the group *i *and segment *j*. This correlation can be tested for significance with a permutation test on the class labels and can also be used to rank the variables according to their importance to discriminate each class. The ranking allows the selection of a group of CNVs which are most relevant for the discrimination. A subsequent unsupervised classification with the selected variables confirms their relevance in population labeling.

### Implementation

The package streamlines CNV segmentation analysis, separated into three major processes: 1) Importing data files containing the normalized *log*_2 _ratio intensities of either Illumina or Affymetrix arrays or any other technology such aCGH, 2) applying GADA and 3) summarizing and visualizing results. These three steps can be performed for an individual array or for multiple samples. In the latter case, the software separates data into different files, one for each sample, allowing an easy way to perform parallel computation.

The functions of the package, their call and arguments are illustrated in detail on the Additional File 1 where specific example applications are described. The file is the user's manual that gives a detailed tutorial for analyzing specific data-sets.

### Importing normalized data

The pipeline analysis of several samples requires a special data management for the input data. Specialized functions like splitDataBeadStudio, for Illumina data, and those in aroma.affimatrix package are used to build the directory structure required by gada. Raw data for *n *subjects stored in a single file is split into *n *files within the subdirectory "rawData", created automatically in the working directory. Loading data into the R session is performed by either setupParGADAIllumina or setupParGADAAffy. An R object of type "setupGADA" is created and taken as input for the segmentation routines. This data handling ensures smooth conversion of array output to suitable input for the segmentation algorithms.

### Segmentation procedure

Segmentation is made of two consecutive algorithms implemented in two separate R-functions within the gada package. The first function (parSBL) uses sparse Bayesian learning (SBL) to discover the most likely positions and magnitudes for a change in copy number, i.e. the breakpoints. The SBL model is governed by a hierarchical Bayesian prior, which is uninformative with respect to the location and magnitude of the copy number changes but restricts the total number of breakpoints. Sensitivity, given by the maximum breakpoint sparseness, is controlled by the hyperparameter *a*. The second function (parBE) is an algorithm that uses a backward elimination (BE) strategy to rank the statistical significance of each breakpoint obtained from SBL. The results from parSBL and parBE are stored in separate files, one for each sample.

### Parallel computation

The package enforces a strict directory structure on the working directory to perform the analysis of multiple samples. However, the only required directory is that containing the raw data. This structure is designed to reduce memory demands and to easily set up simultaneous parallel processes, a main and novel feature of the present implementation.

While the analysis is readily installed to perform single background process, if multiple processors are available, the computing time can be greatly reduced. This facility is implemented with the snow and Rmpi packages http://cran.r-project.org/web/packages/snow. Parallel computation is straight-forward after loading the previous packages.

### Single array

In the case of analyzing a single sample, the file management system is not required. Data can be directly loaded on the R console from a text file containing the *log*_2 _ratios for each probe. Setting up the loaded data into a "setupGADA" object is still required and depends on its original format. This is done with setupGADAIllumina, setupGADAAffy or setupGADAgeneral as previously described. Segmentation follows from the sequential application of the single array functions SBL and BackwardElimination.

### False discovery rate

The critical value *T *required in the BE step is used to establish the final degree of desired sparseness by adjusting the level of FDR. The procedure also requires the specification of the parameter MinSegLen, which determines the minimum number of altered probes in each segment. The two-step strategy (SBL and BE) allows the flexible adjustment of sparseness after breakpoint estimation. Specifically, optimal *T *is found without re-computation of parSBL. Table 1 shows suitable combinations of parameters to achieve desired sensitivity and FDR.

**Table 1:** Parameter selection

(higher sensitivity, higher FDR)	*<-->*	(*a*_*α *_= 0.2*,T > *3)
	*<-->*	(*a*_*α *_= 0.5*,T > *4)
(lower sensitivity, lower FDR)	*<-->*	(*a*_*α *_= 0.8*,T > *5)

Recommended settings of *a *and *T *depending on the desired level of sensitivity and FDR.

**Figure 3:**
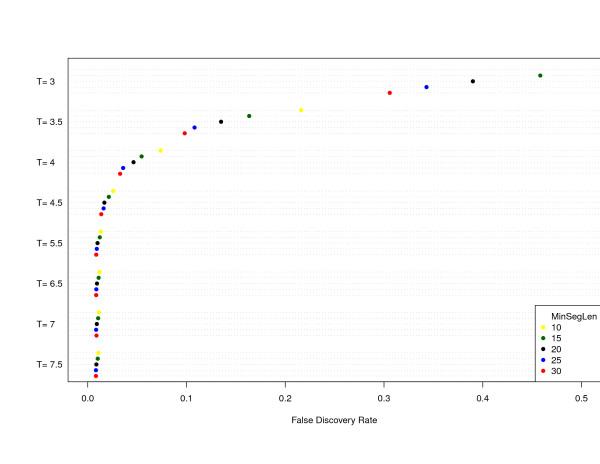
**GADA parameter T as a function of FDR**. The figure shows the increment of T-threshold with False discovery rate (FDR) in a simulation study with *a*_*α *_= 0:2. A selection on T can clearly fix a desired FDR in CNV detection.

A simulation study illustrates the behavior of FDR as function of *T *and MinSegLen. The general simulation model follows [14], however, we have changed the length of the altered and non-altered segments for more realistic matching of non-cancer settings. Figure 3 shows the FDR for different *T *and MinSegLen values. As expected, FDR decreases when *T *or MinSegLen increases.

### Summarizing and displaying segmentation results

The segmentation obtained by GADA returns a collection of segments with breakpoint positions on the most likely locations for copy number change, and segment amplitudes corresponding to the average *log*_2 _ratios of the probes falling between two consecutive breakpoints. The summary function determines which of these ratios correspond to possible changes in copy number, by establishing alteration boundaries. The limits, if not selected by the user, are estimated using the × chromosome of a reference population containing males and females. Segments with amplitudes below (above) the interval limit are reported with a *loss *(*gain*) in copy number. All other segments are considered as non-altered. The result of summary is an R-object of class data.frame that stores the name, chromosome and position of the initial probe of each altered segment, and the number of subject with such alteration.

The R package plotrix is used to display the resulting segments within a given chromosome or across the whole genome. As a data.frame, the output is ready for downstream association analysis implemented on R. Alternatively, they can be exported in BED format and imported into any of the popular genome browsers such as those from *UCSC *and *Ensembl*.

### Multivariate Analysis

The output of the GADA algorithm is easily converted to a matrix of CNVs(columns) and subjects (row) with entries *-*1 (loss), 0 (no-change) and 1(gain). The function getReducedData produces the matrix for all the CNVs across the genome; it also groups and filters the variables. Contiguous variables are grouped together within blocks whose extremes do not differ in more than VarSimil%, and variables (or blocks) with less than 1-subVariation% across subjects are discarded. The parameters are set by the user. If desired, this data reduction can also be performed with other algorithms (i.e. CGHregions), which can be, nevertheless, slower and less flexible for dealing with a large amount of data.

MCA is performed with the general function dudi.acm from ade4, an R package for multivariate analysis. The variable scores, onto the principal component sub-space, can be used as co-variates of an association test to account for population stratification. Specific plots are implemented for displaying such analyses. For identifying a subset of CNVs with the highest prediction power, an MCDA can be performed. More specialized functions, enveloping those of ade4, have been developed for this type of analysis. This functions include discrimin.cnv and rank.variables. The second of these ranks the CNVs according to their correlation onto the population centroids. Variables can be selected by establishing a minimum value for the correlation, assessing their statistical significance with a permutation test, or maximizing the cross-validation accuracy on a train set. Note that the two last options are more computational demanding.

### Association Analysis

Association analysis is implemented for a logistic regression model and a likelihood ratio test (LRT). The function multiCNVassoc repeatedly computes the LRT, comparing a model that includes covariates (e.g., sex, age, population, etc.) with the same model for which the CNV under consideration has been added. The p-value is then computed using a Chi-squared test. This function returns an object of class "multiCNVassoc" that can be printed or plotted using the R generic functions print and plot respectively. We also provide a procedure to deal with multiple comparisons. The function getPvalBH returns the p-values for association corrected by the Benjamini-Hochberg(BH) method. This is a heuristic method that is robust against positive dependence and increasingly conservative as correlation increases. Association tests that need to be corrected for population stratification can incorporate as covariates the scores of the first eigen-values of an MCA.

Note that in this association study CNVs are not treated as simple SNPs. One single probe (not all the probes falling under a CNV) is chosen as a representative of a CNV block. This forms a reduced matrix that is obtained as explained in the previous section. If a complex structure of overlapping CNV segments exists across the samples, multiple alternative segments will be considered for association. Although, the example considered here only covers common CNVs, we anticipate that the approach presented here will be helpful for analyzing more complex scenarios and for developing better association tools. Particularly for rare CNVs, novel association analysis methods are necessary where genes (or DNA regulatory element) are disrupted at different non-overlapping parts.

### Connection with Aroma.Affymetrix

GADA can be called within the Aroma.Affymetrix package http://www.aroma-project.org/, which provides a comprehensive normalization strategy, as used by [6] and an analysis framework for copy number detection and visualization. GADA segmentation tools are specially adapted to the package pipeline, by replacing, within an Aroma.Affymetrix session, the function CbsModel() by our implemented function GadaModel().

## Results and Discussion

In this section we show the results obtained in the main stages of a typical analysis pipeline (the main features of the modules and the type of input/output information they use). The results are illustrated with two sample data sets. The first consist of a set of 8 samples using Illumina Human1M-Duo arrays with 3 M probes. The second dataset consist of 270 lymphoblastoid cell-lines from the HapMap project using Affymetrix SNP 6.0 arrays. A more detailed, step by step, description of these analyses can be followed in Additional file 1.

### Segmentations

The sample data for eight (non HapMap) subjects has over 3 M probes and comes in a BeadStudio format. The first three columns contain name of probe, chromosome and genomic position. Subsequent columns store the (log_2_) ratios for each individual. After splitting into files, loading and setting up, the data was segmented with an *a *= 0.8 and *T *= 8, a choice to optimize FDR. A summary of the output of such segmentation is illustrated as follows:

> allSamples

Summary results for 8 individuals

NOTE: 814 segments with length not in the range 0-Inf bases and with mean log2ratio in the range (-0.24, 0.14) have been discarded

Number of Total Segments:

#   segments   Gains   %   Losses   %

   444   38   8.6   406   91.4

Summary of length of segments:

Min.   1st Qu.   Median   Mean   3rd Qu.   Max.

2169   20510   58600   221200   167700   8547000

Number of Total Segments by chromosome:

   segments   Gains   Losses

Chromosome 1   34   2   32

Chromosome 2   23   2   21

Chromosome 3   16   0   16

Chromosome 4   26   0   26

Chromosome 5   16   2   14

Chromosome 6   74   9   65

Chromosome 7   14   2   12

Chromosome 8   29   2   27

Chromosome 9   12   0   12

Chromosome 10   18   5   13

Chromosome 11   23   3   20

Chromosome 12   10   1   9

Chromosome 13   5   0   5

Chromosome 14   15   1   14

Chromosome 15   12   0   12

Chromosome 16   32   2   30

Chromosome 17   25   2   23

Chromosome 18   9   0   9

Chromosome 19   18   2   16

Chromosome 20   11   0   11

Chromosome 21   6   0   6

Chromosome 22   16   3   13

**Figure 4:**
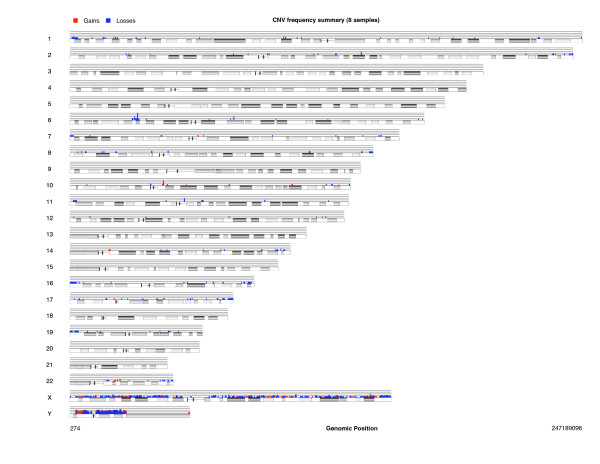
**Probe segmentation across the whole genome**. The figure illustrates the chromosomal positions of copy number alterations found in a sample of 8 individuals. Gains are depicted in red while loses in blue. In this sample alterations are found all across the genome, particularly with more frequency in sexual chromosomes.

**Figure 5:**
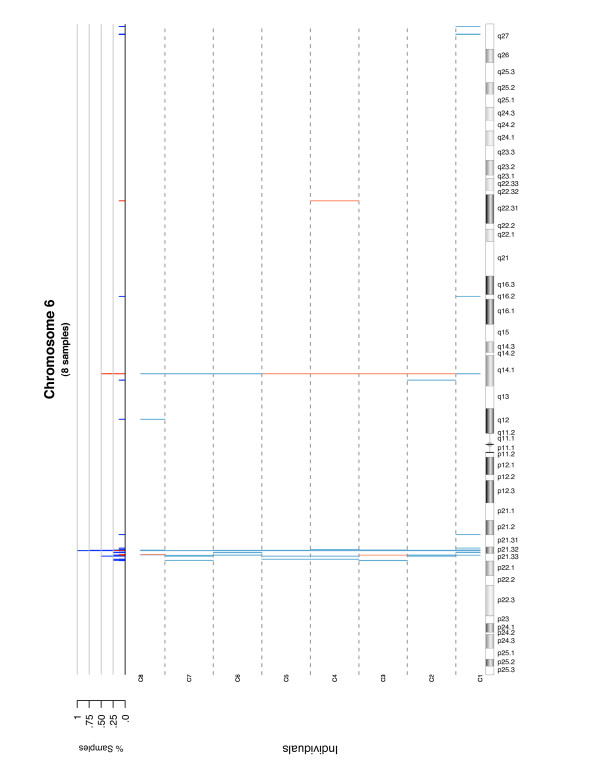
**Probe segmentation in chromosome 6**. Segmentation results for the sample in Figure 4 for chromosome 6 only. The figure shows CNVs of each subject *C_i _*by their chromosomal positions. Gains are shown in red and loses in blue. On the higher row a summary of the group is given by the sample frequency of CNVs on each position.

In this example the limits of the non-altered probes were estimated at (*-*0.24, 0.14) and all segments are considered (length: 0-Inf). If the user is interested in considering only segments in a given range, the argument length.base should be changed indicating their minimum and maximum size. Plots of the segments are readily obtained for the whole genome (Figure 4) or a given chromosome (Figure 5). The processing time for the whole analysis (Mac OS × 10.4.11 2.33 GHz), set up sequentially (not parallel), for eight subjects was approximately 7 min 30 s. Specifically: loading and setting up data: 200.3 s, SBL: 180.1 s and BE: 63.9 s. These times are reduced proportionally to the number of computing cores if the process is parallelized. A previous study [8] showed that the differences in sensitivity and FDR between GADA and CBS are small (*<*3%). However, GADA is 100-fold faster. Here we show that a complete implementation of the algorithm with functions for data handling, segmentation and output writing is still computationally efficient. Given that the core of the segmentation is only two sequential functions, the method is amenable for incorporation as a routine step into association analysis.

The gain/loss ratio of 8.6% versus 91.4% obtained on this 8 samples is different of that of HapMap samples. For the HapMap samples, we obtain 26.2% gain and 73.8% loss CNV segments which is similar to that previously reported by [5]. This higher number of deletions than duplications may be attributed to the greater technical challenge of robustly detecting duplications using oligonucleotide arrays.

### Multivariate analysis

**Figure 6:**
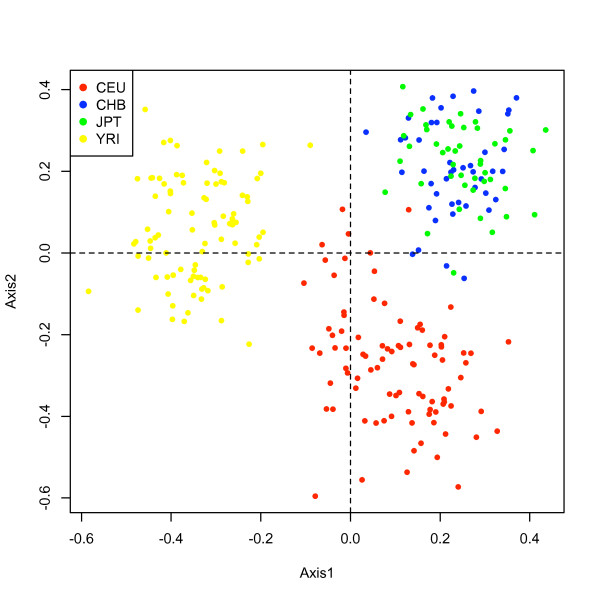
**Subject scores for MCA**. Two first MCA eigen-variables of the HapMap samples. The figure shows that these two variables are sufficient to extract the ancestry differences between the populations, as clear separation between the populations is achieved.

MCA analysis was performed for the HapMap http://www.hapmap.org sample population of 270 subjects (30 trios of CEU and Yoruban each, 45 Han Chinese and 45 Japanese from Tokyo). Data was initially segmented across all chromosomes and a reduced matrix (1465 CNV blocks) was obtained with varSimil = 0.99 and subVariation = 0.90. In Figure 6 we plot the subject scores in the principal axes subspace of a MCA. Labelling each individual with its corresponding group color, we observe that three populations, namely CEU, YRI and CHB-JPT are clearly differentiated. The finer separation between CHB and JPT is not captured at with this unsupervised classification. When analyzing a CNV association study, this loadings can be used to correct for differences in ancestries, as Price et al. [15] have done for SNP data using PCA scores.

**Figure 7:**
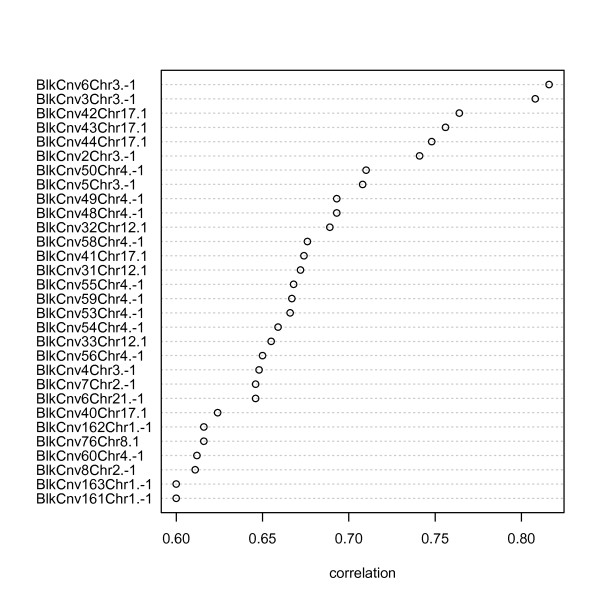
**Variable ranking for MCDA**. CNV blocks (recurrent CNVs) are ranked according to equation 4, that is their maximum correlation across class axes within the principal component subspace. Variables that are more relevant to the discrimination have higher correlations. The figure after the dot refers to relevant level (-1: loss, 0: no gain, 1:gain) of the CNV to discriminate one of the populations.

**Figure 8:**
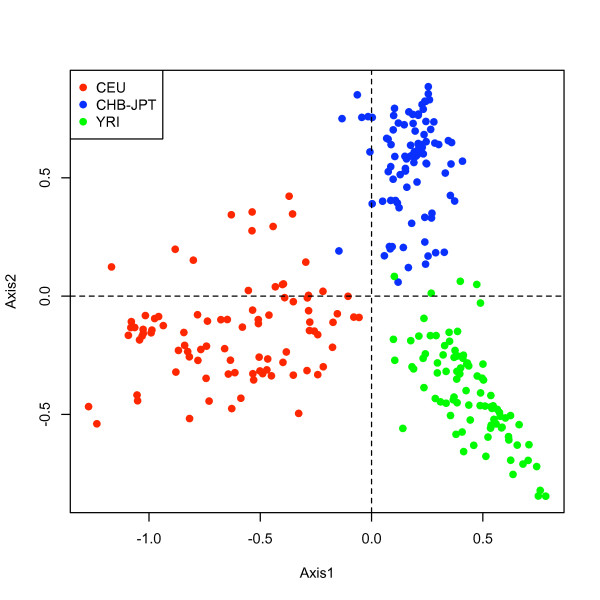
**Subject scores for MCA**. Multivariate analysis run with the highest 87 CNV blocks (*corr > *0:5) ranked with MCDA. The figure shows a high degree of classification for such a reduced set, suggesting that ancestry can be fully accounted with a set of selected CNVs.

**Figure 9:**
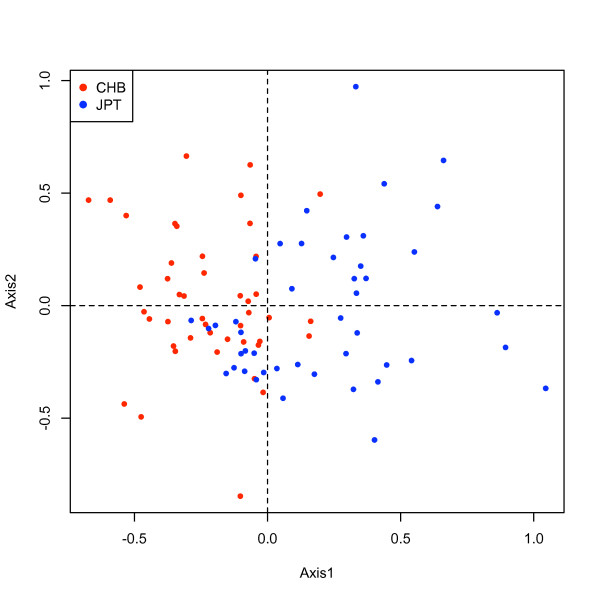
**Subject scores for MCA**. Multivariate analysis for optimal set of variables ranked with MCDA (*corr > *0:5). The figure shows a degree of differentiation between the CHB and JPT populations.

A supervised classification (MCDA) of the samples was performed with discrimin.cnv and followed by rank.variables. The ranking of the variables according to their maximum correlation across group axes is illustrated in Figure 7. We chose 87 variables, which had correlations higher than 0.5, to run an MCA. The results are shown in Figure 8. We observe that this reduced set of variables is able to classify, unsupervised, the populations similarly to the complete set of CNVs. This suggests that population ancestry can be accounted to a high degree with the sampling of very few markers. In addition, we run MCA for the two groups CHB-JPT and chose the optimal variables that revealed a degree of separation between such groups, see Figure 9.

### Association analysis

**Figure 10:**
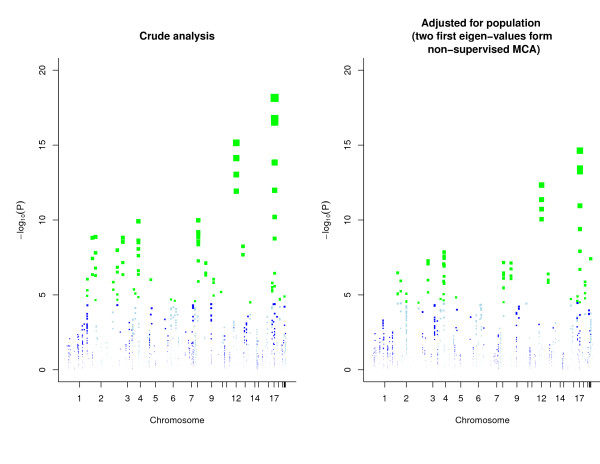
**Association analysis for HapMap individuals using simulated case-control data**. Each panel shows the log_10 _p-value of association. Left panel corresponds to a model including only the CNVs, while the right panel shows the results for a model adjusted for population stratification using the first two eigen-vector from a non-supervised multiple correspondence analysis (MCA). The size of the marks is scaled to the p-value. Those CNVs that pass Bonferroni correction are highlight in green color.

One of the main aims of CNVs studies is to assess association between CNVs and disease. In order to illustrate how to perform this analysis using gada, we have generated case-control data for HapMap samples. We have randomly generated cases and controls with higher proportion of cases for YRI population. Figure 10 is a typical outcome of an association analysis. Here, we illustrate the *-*log_10 _*p *values for association analyses with and without adjusting for population stratification, showing the adequate correction achieved by using MCA.

## Conclusions

We present a package that implements a very flexible pipeline for coupling diverse steps in the analysis of copy number alteration studies. The pipeline integrates in a single R-package the main components of such analysis, such as consecutively coupling normalization, segmentation, recurrent region identification, population stratification analysis and association tests. This unified frame-work and its implementation allow different modules to be easily substituted as new methods or improvements are made.

Some methods incorporate B-allele frequency in the detection of CNVs. Although GADA does not use this information, the algorithm is as precise as CBS and other algorithms that do use such information [8]. It has been shown, however, that the segmentation of the B-allele frequency is informative in detecting mosaicisms as described in [16]. The implementation of GADA in the present pipeline is easily adaptable to this type of analysis and is illustrated in http://groups.google.com/group/gadaproject.

Among its main features, the pipeline enables the import of data from multiple sources (e.g., Affymetrix GTC, Illumina and Aroma.affymetrix). In addition, its segmentation algorithm is very fast, easily parallelized, and its sensitivity can be adjusted quickly. Multiple plotting tools are offered to display results, which can also be exported back into standard formats or used in further R-based analyses. The R-GADA package also integrates new tools for identifying population structure and association analysis. The pipeline is applied to the HapMap samples, illustrating the importance of all the steps in the final association analysis. The package should be specially useful for upcoming CNV association studies which are expected to increase in the number of subjects and probes tested. The segmentation kernel is especially suited for detecting rare and small CNVs, but further efforts are required to develop novel tools that can link these CNVs to underlying functional elements on the DNA sequence.

## Availability and requirements

• Project name: Gada Project

• Project home page: http://groups.google.com/group/gadaproject

• Operating system(s): Platform independent

• Programming language: R 2.9.0

• License: GNU GPL

## Authors' contributions

RPR designed and improved GADA. JRG and RPR implemented the R functions and wrote the article. JRG built the software package. AC implemented the multivariate analysis, analyzed HapMap data and revised the manuscript. All authors read and approved the final manuscript

## Supplementary Material

Additional file 1 **User's Manual**. gada-manual.pdf is the user's guide of gada, where step-by-step segmentation on two sample data sets and the classification of the HapMap groups are described in detail. Click here for file
