# Supplementary material for: R-Gada: a fast and flexible pipeline for copy number analysis in association studies
Source: BMC Bioinformatics. 2010 Jul 16;11:380. doi: 10.1186/1471-2105-11-380 (PMC2915992; doi:10.1186/1471-2105-11-380)
Supplement: Additional file 1 — User's Manual. gada-manual.pdf is the user's guide of gada, where step-by-step segmentation on two sample data sets and the classification of the HapMap groups are described in detail. [file 1471-2105-11-380-S1.PDF]

gada R package: User's manual

Roger Pique-Regi, Alejandro Cáceres and Juan R González

April 7, 2010

1e

Abstract

The gada package is the implementation of a flexible and efficient analysis pipeline to detect genomic copy number alterations from quantitative data. The package can import the raw copy number normalized intensities provided by Illumina BeadStudio, Affymetrix powertools, or any similar format. Probes of different samples are split into separate files and can be analyzed on a standalone workstation or in parallel using a cluster/multicore computer. The speed and accuracy of the genome alteration detection analysis (GADA) approach combined with parallel computing results in one of the fastest and most accurate methods available. GADA is especially suitable to extract copy number alterations (CNAs) on genomewide studies that utilize high density arrays of millions of markers to sample hundreds of subjects.

Contents

1 Installation **2**

2 Analysis of a single array **2**
2.1 Importing and preparing array data, the `setupGADA` class. . . . 2
2.1.1 Creating a `setupGADA` object using `setupGADAgeneral` 2
2.1.2 Creating a `setupGADA` object for Illumina or Affymetrix array 3
2.2 Summarizing data 4
2.3 Copy number segmentation with `SBL` and `BackwardElimination` 7

3 Multiple array analysis **12**
3.1 Raw data 12
3.1.1 Importing a collection of Illumina array data 12
3.1.2 Importing a collection of Affymetrix array data 14
3.2 Segmentation procedure 14
3.2.1 Paralell segmentation 16
3.3 Summaryzing results 16

4 Multivariate analysis of segmented data **21**

5 Association analysis **24**

6 Exporting data from Illumina and Affymetrix platforms to gada **26**
6.1 Exporting data from Bead Studio 26
6.2 Exporting data from Affymetrix genotyping console (GTC) 31
6.3 Exporting data from Affymetrix power tools (APT) 32

7 Tutorial session with Affymetrix data **34**
7.1 Analyzing a single Affymetrix array 34
7.2 Analyzing a collection of 90 Affymetrix arrays 38

8 Connection with Aroma.Affymetrix **45**

1 Installation

The R scripts and package described in this manual are accessible at `http://groups.google.com/group/gadaproject`, where the `.tar.gz` source file can also be found.

From the R command line, `gada` is installed using:

```
> install.packages("gada_0.8-0.tar.gz",repos=NULL)
```

Another option is to install the package from CRAN (not yet available)

```
> install.packages("gada")
```

Then, the package is loaded, by typing:

```
> library(gada)
```

2 Analysis of a single array

2.1 Importing and preparing array data, the setupGADA class.

The first step for the analysis is to prepare a **setupGADA** object that encapsulates the array hybridization intensities and other information such as the marker position in the genome and the genotype, in case of SNP markers. If data is already loaded on `R`, the object is obtained with the function **setupGADAgeneral** . Otherwise, data can be loaded with **setupGADAIllumina** from the text files exported by Illumina BeadStudio, or with **setupGADAaffy** for files obtained with Affymetrix Genotyping Console (Section 2.1.2). These functions can also be used with other array platforms of similar output format.

2.1.1 Creating a setupGADA object using setupGADAgeneral

If data is already available in `R` then **setupGADAgeneral()** directly creates a **setupGADA** object, like in the example:

```
> ## Simulated data
> set.seed(123456)
> cn<-rep(c(rep(1,1E5-100),rep(1,100),rep(1,1E5)),4) #Underlying copy number
> arrayData<-rnorm(length(cn),mean=(log2(cn)-1),sd=1) #Simulated array
> dataSim<-setupGADAgeneral(arrayData) #Prepared setupGADA object
> dataSim
Object of class 'setupGADA' (log.ratio data)
--------------------------------------------
  Number of probes: 800000 (0 missing values)

  Number of probes by chromosome:
    No genetic information available
```

where we use a simulated sample. Annotation data, if available, can also be added through the argument `gen.info` as a data.frame. The following format is required:

```
        probe chr    pos
1  rs12354060   1  10004
2   rs6650104   1 554340
3  rs12184279   1 707348
4  rs12564807   1 724325
5   rs3115860   1 743268
6   rs7515489   1 758845
7  rs17160939   1 773886
8  rs12086311   1 798632
9   rs4475691   1 836671
10 rs28705211   1 890368
...
```

```
> gen.info <- data.frame( probe=paste("id",1:length(cn),sep=""),
+            chr=c(rep(1,2E5),rep(2,2E5),rep(3,2E5),rep(4,2E5)),
+            pos=rep(1:(length(cn)/4),4)*10)
> ## setupGADA object with annotation information
> dataSim<-setupGADAgeneral(arrayData,gen.info=gen.info)
> dataSim
Object of class 'setupGADA' (log.ratio data)
--------------------------------------------
  Number of probes: 800000 (0 missing values)

  Number of probes by chromosome:
     1      2      3      4
200000 200000 200000 200000
```

2.1.2 Creating a `setupGADA` object for Illumina or Affymetrix array

Data exported from Illumina using BeadStudio tool, or Affymetrix using Affymetrix genotyping console (GTC) or Affymetrix power tools (APT) are loaded using `setupGADAIllumina()` or `setupGADAAffy()` functions, respectively. Sections 6.1 and 6.2 illustrates how to export data from both technologies. In either case, data must be arranged in the following format:

- 1st column: probe
- 2nd column: chromosome
- 3rd column: genomic position
- 4th column: ... other information
- ...
- jth column: ... log2ratio
- ...
- kth column: ... other information

Two example files are provided for further detail. The first one corresponds to an Illumina data example:

```
Name Chr Position GType Allele Freq Log R Ratio
rs1000050 1 161003087 AB 0.4960448 -0.1494603
rs1000073 1 155522020 AB 0.4824853 0.00509767
rs1000313 1 15278076 AA 0 -0.1521843
rs1000476 1 58694104 AA 0.001480275 0.09277323
rs1000533 1 166549115 AB 0.5048196 -0.002900129
rs1000543 1 242254223 BB 1 -0.01190711
rs1000730 1 230030224 AB 0.5039815 0.06360321
rs1000731 1 230030114 BB 0.998738 0.05265531
rs1000997 1 15998548 AB 0.5515608 0.03962962
rs1001149 1 150775186 BB 1 0.06456274
rs1001160 1 76131179 AA 0 -0.0726123
rs1001193 1 145633001 AA 0 -0.1115284
```

and can be downloaded with:

```
> download.file("http://www.creal.cat/jrgonzalez/GADA/dataIllumina.txt",
+               "./dataIllumina.txt")
trying URL 'http://www.creal.cat/jrgonzalez/GADA/dataIllumina.txt'
Content type 'text/plain' length 24698671 bytes (23.6 Mb)
opened URL
==================================================
downloaded 23.6 Mb
```

The second example is a sample obtained from the Affymetrix plataform:

```
$ head -500 NA06985_GW6_C.MyTest.CN5.CNCHP.txt

#Comments
....
#Comments
#Comments
ProbeSetName    Chromosome      Position        CNState Log2Ratio       SmoothSignal    LOH     Allele Difference
CN_473963       1       51586   2       -0.257667       1.054558        nan     nan
CN_473964       1       51659   2       -0.264712       1.054389        nan     nan
CN_473965       1       51674   2       -0.043675       1.054354        nan     nan
CN_473981       1       52771   2       -0.402939       1.051817        nan     nan
CN_473982       1       52788   2       0.134605        1.051777        nan     nan
CN_497981       1       62627   2       -0.006367       1.029375        nan     nan
CN_502615       1       75787   2       -0.677508       1.000571        nan     nan
CN_502613       1       75849   2       -0.343111       1.000438        nan     nan
CN_502614       1       76175   0       -1.440673       0.999744        nan     nan
CN_502616       1       76192   0       -2.477916       0.999708        nan     nan
CN_502843       1       88453   2       -0.135097       0.974336        nan     nan
CN_466171       1       218557  2       -0.030157       1.597003        nan     nan
CN_468414       1       218926  2       -0.475484       1.597018        nan     nan
CN_468412       1       219009  2       -0.045742       1.597021        nan     nan
CN_468413       1       219024  2       -0.050614       1.597022        nan     nan
...
```

```
> download.file("http://www.creal.cat/jrgonzalez/GADA/NA12248_GW6_C.MyTest.CN5.CNCHP.txt",
                "./NA12248_GW6_C.MyTest.CN5.CNCHP.txt")
```

Both `setupGADAIllumina()` or `setupGADAAffy()` functions have the same arguments, and are called in a similar manner

```
> dataIllumina<-setupGADAIllumina(file="dataIllumina.txt",log2ratioCol=5,NumCols=6)
Read 3367818 items
```

and

```
> dataAffy <- setupGADAaffy(file="NA12248_GW6_C.MyTest.CN5.CNCHP.txt",
                            NumCols=8,log2ratioCol=5)
Read 14507536 items
```

where `file` indicates either the url or the path to the file which contains the data (`file="dataIllumina.txt"`), `log2ratioCol` informs which column contains the $\log_2$ ratio instensities, and `NumCols` gives the number of columns the file has. Note that results exported from APT tools (Section 6.3) should be specified with `NumCols=8`, `log2ratioCol=5`. Whereas those of Affymetrix Genotyping Console (Section 6.2), are `NumCols=4` and `log2ratioCol=4`. The argument `sort` is equal to TRUE by default, in order to ensure that the data is correctly arranged by chromosomal position. However, if data is already ordered, setting `sort=FALSE` will reduce computing time.

```
# Not run
dataIllumina2<-setupGADAIllumina(file="dataIllumina.txt", log2ratioCol=6,
                                 NumCols=6, sort=FALSE)
# End not run
```

Other arguments such as `saveGenInfo` or `orderProbes` are used internally, and it is not recommended to change them.

2.2 Summarizing data

Imported data is summarized entering the name of the object of class `setupGADA` or using the generic method `print`.

```
Object of class 'setupGADA' (Illumina data)
-------------------------------------------
  Number of probes: 561303 (118 missing values)

  Number of probes by chromosome:
    1     2     3     4     5     6     7     8     9    10    11    12    13
42075 45432 37768 33705 34649 36689 30170 31880 26874 29242 27272 27143 20914
```

```
   14    15    16    17    18    19    20    21    22     X     Y
18429 16625 16870 14341 16897  9501 14269  8251  8462 13835    10
```

Figure 2.2 shows the log-ratio intensities and obtained with `plotRatio`. This function has several arguments that can be used to draw different types of plots. This visualization tools require the package `plotrix` which is available from CRAN. It can be installed by typing `install.packages("plotrix")`. By default `plotRatio` produces an output like Figure 2.2 which displays the entire genome.

```
> plotRatio(dataIllumina)
Loading required package: plotrix
```

Intensities along the karyotype of a single chromosome (e.g. chr 12, Figure 2.2) are displayed with

```
> plotRatio(dataIllumina,chr=12)
```

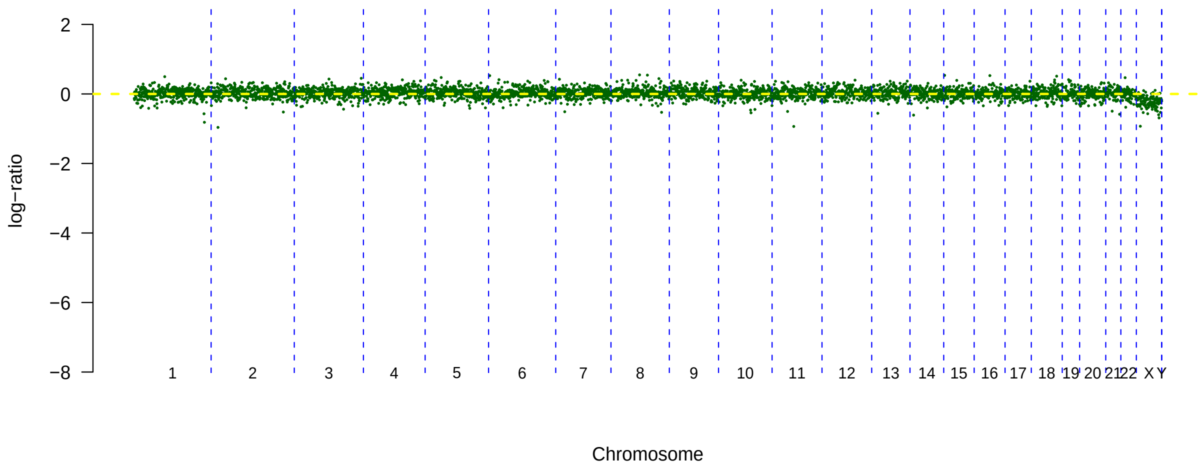

Figure 1: Illumina log2ratio intensities by chromosome

All plots that we have previously illustrated can be saved as a encapsulated postscript (eps) file using the `postscript` R function:

```
> postscript(file="log_intensities.eps")
> plotRatio(dataIllumina, postscript=TRUE)
> dev.off()
```

Visualizing all the probes on a single plot may generate an unnecessarily big file given the high resolution of the array platforms. We can reduce the number of points in the plot modifying the argument `num.points`.

```
> postscript(file="log_intensities.eps")
> plotRatio(dataIllumina, postscript=TRUE, num.points=50000)
> dev.off()
```

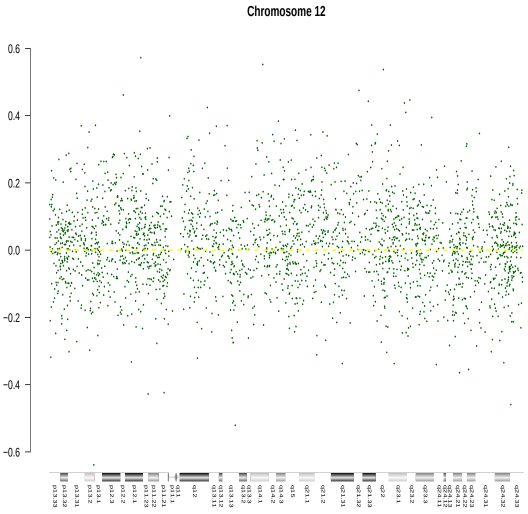

Figure 2: Illumina log2ratio intensities for chromosome 12

2.3 Copy number segmentation with SBL and BackwardElimination

The segmentation procedure is divided in two steps as described in [2]. The first step fits a sparse Bayesian learning (SBL) model and finds the most likely candidate breakpoints for the copy number state. The second step, implements a backward elimination (BE) procedure to remove sequentially the least significant breakpoints estimated by the SBL model, allowing a flexible adjustment of the False Discovery Rate (FDR).

The first step is implemented on the SBL procedure

```
> step1<-SBL(dataIllumina, estim.sigma2=TRUE)
    The estimated sigma2 = 0.01411465
```

and requires the setupGADA object, e.g. dataIllumina in previous section. The SBL is controled by two parameters: 1) the array noise level σ^2, and 2) the sparseness hyperparameter a_α. The array noise level σ^2 is estimated automatically by the algorithm by setting estim.sigma2=TRUE. If σ^2 is known a priori it can be set manually with the assignment sigma2=σ^2. The sparseness hyperparameter a_α (i.e. aAlpha) controls the SBL prior distribution which is uninformative about the location an amplitude of the CNA breakpoints but imposes a penalty on the number of CNA breakpoints. A higher a_α implies that less breakpoints are expected a priori and results with fewer true CNA detected, yet fewer false positives. An efficient adjustment of the trade-off between sensitivity and FDR is performed with a backward elimination (BE) procedure, using a high sensitivity of $a_\alpha = 0.2$, set up by default aAlpha=0.2 .

The second step, a backward elimination procedure implemented in BackwardElimination, is used to adjust the FDR,

```
step2<-BackwardElimination(step1,T=4.5,MinSegLen=3)
```

where the T argument is the critical value of the BE algorithm for the statistical score t_m, associated to brake-point m. That is, t_m lower than T are discarded. The score t_m is the difference between the sample averages of the probes falling on the left and right segment, divided by a pooled estimation of the standard error. Asymptotically, when the number of probes on the right and left segments are very large the distribution of score will converge to a standard normal distribution, i.e. $\mathcal{N}(0, 1)$.

The argument MinSegLen can be used to limit the minimum number of probes each CNA segment must contain. We recommend using MinSegLen=3 (default) to eliminate false detections due to extreme outliers.

The following settings on a and T are recommended depending on the desired sensitivity and FDR:

(higher sensitivity , higher FDR)	$<-->$	($a_\alpha = 0.2$,$T > 3$)
	$<-->$	($a_\alpha = 0.5$,$T > 4$)
(lower sensitivity , lower FDR)	$<-->$	($a_\alpha = 0.8$,$T > 5$)

The print generic function gives the user the following information for each step:

```
> step1
Sparse Bayesian Learning (SBL) algorithm
sigma2 = 0.0141
-------------------------------------------------------------
   chromosome discontinuities numit    tolerance
1           1            1811  1434 9.884051e-09
2           2            2013   917 9.955136e-09
3           3            1653   660 9.958175e-09
4           4            1558  2521 9.791328e-09
5           5            1551   581 9.990603e-09
6           6            1671  1968 9.951486e-09
7           7            1373   762 9.854208e-09
8           8            1330  1252 9.893025e-09
9           9            1148   900 9.599827e-09
```

```
10          10            1233   791 9.993864e-09
11          11            1165  1374 9.927208e-09
12          12            1084   645 9.877164e-09
13          13             976   697 9.869698e-09
14          14             816   705 9.919774e-09
15          15             661   423 9.806253e-09
16          16             643   571 6.404507e-09
17          17             478  3302 9.226409e-09
18          18             751   468 9.694729e-09
19          19             383   849 9.933759e-09
20          20             521  1362 9.955395e-09
21          21             374   716 9.933086e-09
22          22             359   440 9.927054e-09
23           X            1472  1926 9.949394e-09
24           Y               1    50 8.340744e-09
```

```
> step2
Sparse Bayesian Learning (SBL) algorithm
SBL and Backward Elimination with T=4.5 and minimum length size=3
sigma2 = 0.0141
------------------------------------------------------------
   chromosome discontinuities
1           1              36
2           2              30
3           3              39
4           4              22
5           5              28
6           6              26
7           7              29
8           8              33
9           9              24
10         10              16
11         11              41
12         12              23
13         13              17
14         14              17
15         15              11
16         16               9
17         17               8
18         18              16
19         19               3
20         20              10
21         21               6
22         22               4
23          X              43
24          Y               0
```

The `SBL` function returns the number of discontinuities for each chromosome, the number of iterations and the tolerance given to the SBL algorithm to converge. The `BackwardElimination` function gives the number of segments for each chromosome adjusted by the parameter `T` and the minimum number of consecutive altered probes in the argument `MinSegLen`. We would like to highlight the advantage of using a two step approach. We can flexibly adjust T (remove or add breakpoints that will follow in significance) without having to fit the entire SBL model again. As T and MinSegLen increase the number of CNA breakpoints decreases.

Finally, the altered segments defined between the modeled breakpoints are reported using the `summary` R method. We classify the segments as gain and losses using a simple threshold on the segment mean

amplitude (i.e. MeanAmp).

```
> summary(step2)
----------------------------------------
Sparse Bayesian Learning (SBL) algorithm
Backward Elimination procedure with T=4.5 and minimun length size=3
 Number of segments =  516
 Base Amplitude of copy number 2: chr 1:22:0.0174, X=-0.221, Y=0.0275
 Gains (1) and Losses (-1) with respect Base Amplitude
----------------------------------------
     IniProbe  EndProbe LenProbe      MeanAmp chromosome State
1      742429  25178194     4906 -0.033250509          1    -1
2    25179149  25264714       41 -0.151262862          1    -1
3    25264951  41720331     2578 -0.037778763          1    -1
4    41725184  41726332        3 -0.355878820          1    -1
5    41727031  50481809     1289 -0.023630115          1    -1
6    50489793  52876849      200  0.029492261          1     1
7    52878645  61404617     1970 -0.020525958          1    -1
9    70694440  80309386     1612  0.036965666          1     1
10   80309805  80320023        4  0.327258580          1     1
11   80329403  82192926      431  0.045561250          1     1
12   82193305  97356749     2802  0.012820850          1     1
13   97357234 107627389     1695  0.043190020          1     1
15  110070351 110984890      244 -0.054825800          1    -1

...

496 115538986 115553916        4  0.133537435          X     1
498 125716562 125719857        3 -0.591493700          X    -1
500 126565408 126586900        4 -4.768452000          X    -1
504 128403153 128461071        7 -0.012350443          X     1
506 130307929 130873100       60  0.346408771          X     1
508 137853309 138032698       20 -0.052194362          X     1
510 144181999 144968405      111 -0.292001868          X    -1
512 147115894 147246249       10 -0.411471160          X    -1
514 154545424 154871186        7 -0.018561945          X     1
```

The function estimates the reference ratio corresponding to two copy numbers ('Base Amplitude of copy number 2 in the output) computing the median intensity along the autosomal genome. This value can also be manually specified using `summary(step2,BaseAmp=0)`. After that, the segment mean amplitude `MeanAmp` is normalized by subtracting the reference ratio of two copy numbers in order to take into account differences between arrays with respect to uncontrolled factors, like amount of DNA, different laboratories, etc. The segments are then classified as Gain (`State=1`), Loss (`State=-1`), or Neutral (`State=0`) depending on whether the segment mean amplitude is above, below, or non-significantly different than `BaseAmp`. Only the segment with significant deviations, gains or losses, are reported by `summary`.

`plotRatio` shows the log-ratio intensities as well as the segments obtained after backward elimination procedure (Figure 2.3).

```
> plotRatio(step2)
----------------------------------------
Sparse Bayesian Learning (SBL) algorithm
Backward Elimination procedure with T=4.5 and minimun length size=3
 Number of segments =  515
 Base Amplitude of copy number 2: chr 1:22:0.0174, X=-0.221, Y=0.0275
```

This plot can also be obtained chromosome wise. As an example, Figure 2.3 shows the intensities and segments found after applying the backward elimination procedure in chromosome 12.

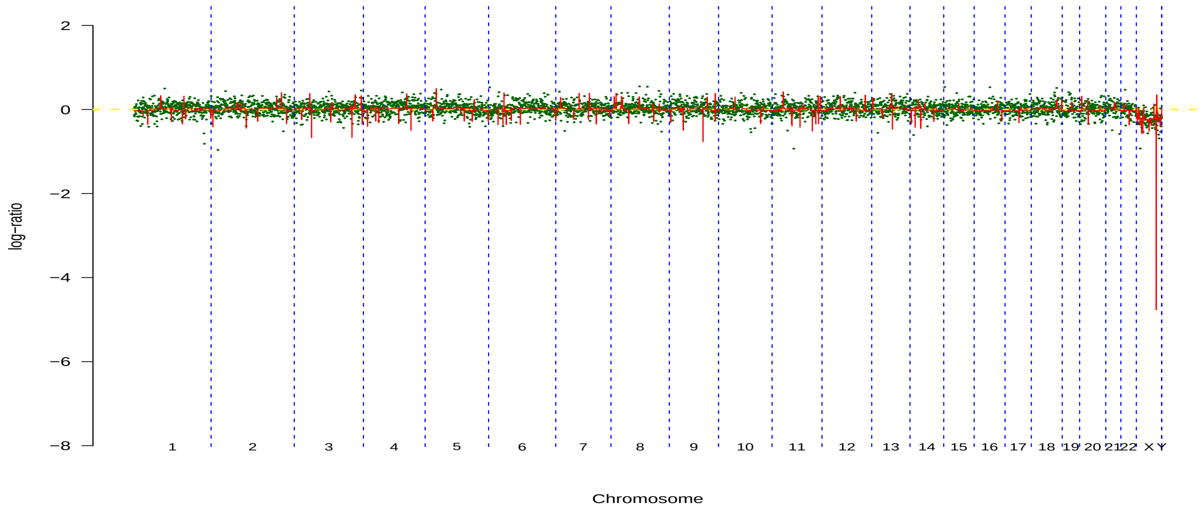

Figure 3: log-ratio intensities and segments for the entire genome

```
> plotRatio(step2, chr=12)
---------------------------------------
Sparse Bayesian Learning (SBL) algorithm
Backward Elimination procedure with T=4.5 and minimun length size=3
 Number of segments =  515
 Base Amplitude of copy number 2: chr 1:22:0.0174, X=-0.221, Y=0.0275
```

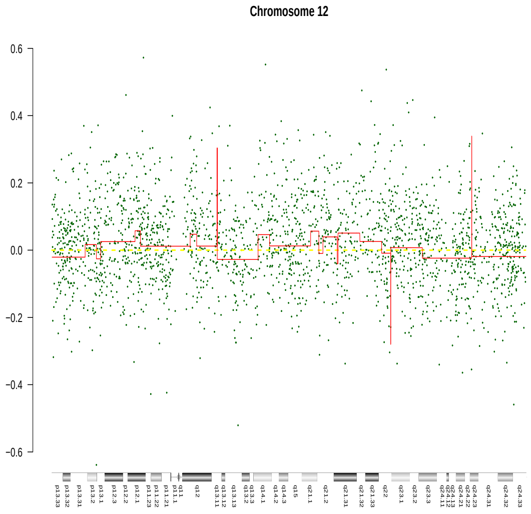

Figure 4: log-ratio intensities by chromosome and break-points for chromosome 12

3 Multiple array analysis

The package enforces a strict directory structure on the working directory to perform the analysis of multiple samples. However, the only required directory to be set up by the user is that containing the raw data. This is an example of how folders are organized after analysis is completed

```
|-- exampleBeadStudio.txt
|-- rawData
|   |-- sample.C1
|   |-- sample.C2
|   |-- sample.C3
|   |-- sample.C4
|   |-- sample.C5
|   |-- sample.C6
|   |-- sample.C7
|   `-- sample.C8
|-- SBL
|   |-- allSegments
|   |-- gen.info.Rdata
|   |-- genomicInfo
|   |-- sbl1
|   |-- sbl2
|   |-- sbl3
|   |-- sbl4
|   |-- sbl5
|   |-- sbl6
|   |-- sbl7
|   |-- sbl8
|   |-- segments1
|   |-- segments2
|   |-- segments3
|   |-- segments4
|   |-- segments5
|   |-- segments6
|   |-- segments7
|   |-- segments8
|   |-- setupGADA1
|   |-- setupGADA2
|   |-- setupGADA3
|   |-- setupGADA4
|   |-- setupGADA5
|   |-- setupGADA6
|   |-- setupGADA7
|   `-- setupGADA8
```

3.1 Raw data

The *rawData* directory must contain all data files corresponding to each individual from a particular assay. Each data file must be organized as described in Section 2.1.

3.1.1 Importing a collection of Illumina array data

The user may have all information in a unique file as indicated in Section 6.1. In this case, the user can obtain individual files from `gada` by using `splitDataBeadStudio` function. To illustrate how to split the file into subject files, we use the following files available at

```
> download.file("http://www.creal.cat/jrgonzalez/GADA/exampleBeadStudio.txt","./exampleBeadStudio.txt")
```

Notice that the three first columns of this file must contain annotation data. This information is required and it includes the name of probe, the chromosome and the genomic position. The other columns correspond to each individual. Note however that the information can be variable, depending on the information we have obtained from BeadStudio. In this example, we saved the log2ratio and the B-allele frequency.

```
Name Chr Position C1 Log R Ratio C1 B Allele Freq C2 Log R Ratio C2 B Allele Freq C3 Log R Ratio C3 B Allele Freq C4 Log R Ratio C4 B Allele Freq C5 Log R Ratio C5 B Allele Freq
C6 Log R Ratio C6 B Allele Freq C7 Log R Ratio C7 B Allele Freq C8 Log R Ratio C8 B Allele Freq
rs758676 7 12878632 0.1134 0.5215 -0.0312 1.0000 -0.0098 1.0000 0.0442 1.0000 -0.1815 0.9942 0.1144 0.4990 -0.5641 0.0000 -0.0488 1.0000
rs3916934 13 103143536 0.2099 0.0014 0.1669 0.5361 -0.2143 0.0062 0.0371 0.9955 -0.3281 0.0048 -0.2505 0.5249 -0.2122 0.5427 0.0262 0.9912
rs2711935 4 38838852 0.0443 0.0000 0.0972 0.5094 0.1467 0.0109 0.1192 0.4951 0.0490 0.4725 -0.0704 0.0001 -0.1707 0.4987 0.2628 0.5125
rs17126880 1 64922104 0.0659 0.9888 0.0917 1.0000 -0.0008 0.9986 -0.0442 0.9959 0.0766 1.0000 -0.0343 0.9949 0.0454 0.9989 -0.0398 0.9930
rs12831433 12 4995220 -0.0072 0.0043 0.0782 0.0006 0.0927 0.5063 0.2230 0.5282 -0.0317 0.0026 0.2575 0.9929 0.1471 0.0000 -0.0219 0.0066
```

```
> splitDataBeadStudio("exampleBeadStudio.txt",Samples=8,NumCols=5)

Splitting data from BeadStudio ...

Obtaining Ratio Intensity files ...
NOTE: individual files will be written to 8 files with name as indicated in header of input file
Obtaining Ratio Intensity files ... done
```

`splitDataBeadStudio` has two parameters `Samples` and `NumCols`. The first one indicates the number of individuals analyzed (in our case 8 samples). The argument `NumCols` gives the number of columns, considering the three first columns that contain the annotation data. As we have information about log2ratio and B-allele frequency, the argument `NumCols` is set equal to 5.

Once individual files are available, we then import a collection of Illumina array data with

```
> myExample<-setupParGADAIllumina(log2ratioCol=4, NumCols=5)

Creating object with annotation data ...
Read 3218460 items
Creating object with annotation data ...done

Creating objects of class setupGADA for all input files...
  Applying setupGADAIllumina for 8 samples ...
  Importing array:  sample.C1 ... Read 5364100 items
   Array # 1 ...done
  Importing array:  sample.C2 ... Read 5364100 items
   Array # 2 ...done
  Importing array:  sample.C3 ... Read 5364100 items
   Array # 3 ...done
  Importing array:  sample.C4 ... Read 5364100 items
   Array # 4 ...done
  Importing array:  sample.C5 ... Read 5364100 items
   Array # 5 ...done
  Importing array:  sample.C6 ... Read 5364100 items
   Array # 6 ...done
  Importing array:  sample.C7 ... Read 5364100 items
   Array # 7 ...done
  Importing array:  sample.C8 ... Read 5364100 items
   Array # 8 ...done
  Applying setupGADAIllumina for 8 samples ... done
Creating objects of class setupGADA for all input files... done
```

This function calls repeatedly the function `setupGADAIllumina`, so the arguments `log2ratioCol` and `NumCols` are passed through the function `setupGADAIllumina`; see section 2.1. Other arguments for `setupGADAIllumina` can also be set from this function. The function saves an object of class `setupGADA` for each sample in the directory *SBL*. The function returns an object of class `parGADA`, which allows the process to be resumed later. An object of class `parGADA` contains this information.

```
> myExample
[1] "/home/jrgonzalez/CREAL/GADA"
```

```
attr(,"class")
[1] "parGADA"
attr(,"type")
[1] "Illumina"
attr(,"labels.samples")
[1] "C1" "C2" "C3" "C4" "C5" "C6" "C7" "C8"
attr(,"Samples")
[1] 8
```

This object is throughly used in the analysis and plotting procedures. For instance, a plot for individual 4 with log2ratio intensities can be obtained with

```
> # plot for sample #4
> plotRatio(myExample,Sample=4)
```

and the same plot including the segments is obtained via:

```
> # plot for sample #4 with segments
> plotRatio(myExample,Sample=4,segments=TRUE)
```

It is recommended to save this object to continue performing the analysis in case of R need to be restarted

```
> save(myExample,file="myExample.Rdata")
```

3.1.2 Importing a collection of Affymetrix array data

The function setupParGADAaffy should be used in the case of having data from Affymetrix. The performance of this function is similar to the previous one.

```
> myExampleAffy <- setupParGADAaffy(log2ratioCol=4,NumCols=4);

Creating objects of class setupGADA for all input files...
  Applying setupGADAaffy for 90 samples ...
  Importing array:  NA06985_GW6_C.CN5.CNCHP.myAffyData.txt ... Read 7253768 items
   Array # 1 ...done
  Importing array:  NA06991_GW6_C.CN5.CNCHP.myAffyData.txt ... Read 7253768 items
   Array # 2 ...done

...

  Importing array:  NA12892_GW6_C.CN5.CNCHP.myAffyData.txt ... Read 7253768 items
   Array # 90 ...done
  Applying setupGADAaffy completed succesfully.
```

3.2 Segmentation procedure

Once raw data is imported as objects of class setupGADA, we can perform segmentation procedure for all individuals one by one. The procedure for analyzing Illumina and Affymetrix data is the same. Here, we use the example of Illumina data to illustrate how to perform parallel segmentation procedure. The Appendix shows an example for Affymetrix data.

To perform segmentation procedure for multiple arrays, we use the function parSBL that repeatedly calls the function SBL. The syntax is similar to those used in the function SBL:

```
> parSBL(myExample, estim.sigma2=TRUE, aAlpha=0.8)
Creating SBL directory ...done
Retrieving annotation data ...done
Segmentation procedure for 8 samples ...
   Array # 1 ...    The estimated sigma2 = 0.02312321
   Array # 1 ...done
   Array # 2 ...    The estimated sigma2 = 0.01455486
   Array # 2 ...done
   Array # 3 ...    The estimated sigma2 = 0.01264846
```

```
   Array # 3 ...done
   Array # 4 ...    The estimated sigma2 = 0.01334702
   Array # 4 ...done
   Array # 5 ...    The estimated sigma2 = 0.01252028
   Array # 5 ...done
   Array # 6 ...    The estimated sigma2 = 0.02356903
   Array # 6 ...done
   Array # 7 ...    The estimated sigma2 = 0.02532079
   Array # 7 ...done
   Array # 8 ...    The estimated sigma2 = 0.02927364
   Array # 8 ...done
Segmentation procedure for 8 samples ...done
```

In this case we perform the segmentation procedure for all samples in the folder SBL that have been imported as a `setupGADA` objects. It is possible to perform segmentation procedure for a subset of individuals, by using the argument `Samples` as following

```
> # Not run
> parSBL(myExample, Samples=c(4,8), estim.sigma2=TRUE)
> # End not run
```

The SBL result for each array is stored in a directory called *SBL*. Notice that in this case the argument `Samples` is a vector pair indicating the first and the last individual to be analyzed. Therefore, if the process is stopped for any reason, the analysis for the subjects left out is easily resumed.

Similarly, the backward elimination (BE) for multiple individuals is implemented in the function `multiBE`.

```
> parBE(myExample,T=8, MinSegLen=8)
Retrieving annotation data ...done
Backward elimination procedure for 8 samples ...
   Array # 1 ... ---------------------------------------
Sparse Bayesian Learning (SBL) algorithm
Backward Elimination procedure with T=8 and minimun length size=8
 Number of segments =  878
 Base Amplitude of copy number 2: chr 1:22:0.0274, X=-0.0433, Y=0.1369
   Array # 2 ... ---------------------------------------
Sparse Bayesian Learning (SBL) algorithm
Backward Elimination procedure with T=8 and minimun length size=8
 Number of segments =  151
 Base Amplitude of copy number 2: chr 1:22:-0.0097, X=-0.0844, Y=-0.0637
   Array # 3 ... ---------------------------------------
Sparse Bayesian Learning (SBL) algorithm
Backward Elimination procedure with T=8 and minimun length size=8
 Number of segments =  208
 Base Amplitude of copy number 2: chr 1:22:0.0118, X=-0.0377, Y=-1.0436
   Array # 4 ... ---------------------------------------
Sparse Bayesian Learning (SBL) algorithm
Backward Elimination procedure with T=8 and minimun length size=8
 Number of segments =  542
 Base Amplitude of copy number 2: chr 1:22:-0.0054, X=0.3908, Y=-4.3155
   Array # 5 ... ---------------------------------------
Sparse Bayesian Learning (SBL) algorithm
Backward Elimination procedure with T=8 and minimun length size=8
 Number of segments =  560
 Base Amplitude of copy number 2: chr 1:22:-0.0056, X=0.3985, Y=-4.1761
   Array # 6 ... ---------------------------------------
Sparse Bayesian Learning (SBL) algorithm
Backward Elimination procedure with T=8 and minimun length size=8
 Number of segments =  82
```

```
 Base Amplitude of copy number 2: chr 1:22:0.0023, X=-0.0708, Y=0.0727
   Array # 7 ... ---------------------------------------
Sparse Bayesian Learning (SBL) algorithm
Backward Elimination procedure with T=8 and minimun length size=8
 Number of segments =  260
 Base Amplitude of copy number 2: chr 1:22:-0.0989, X=0.3088, Y=-3.4627
   Array # 8 ... ---------------------------------------
Sparse Bayesian Learning (SBL) algorithm
Backward Elimination procedure with T=8 and minimun length size=8
 Number of segments =  100
 Base Amplitude of copy number 2: chr 1:22:-0.0159, X=-0.1274, Y=-0.0553
Backward elimination procedure for 8 samples ...done
```

The function stores the segments in the directory *SBL*. The arguments are the same as those used in the function `BackwardElimination` previously described.

3.2.1 Paralell segmentation

We have programmed `setupParGADAIllumina`, `setupParGADAaffy`, `parSBL` and `parBE` functions to allow the user to parallelize, in few steps, the analysis when multiple processors are available. This has been implemented using the `snow` package. After loading the required packages `snow` and `Rmpi`

```
> library(snow)
> library(Rmpi)
```

we create the cluster (`cl`). We use the instruction

```
> cl<-makeCluster(8,type="MPI")
        8 slaves are spawned successfully. 0 failed.
```

Further examples, including how to connect more than one workstation, can be found in http://www.sfu.ca/ sblay/R/snow.htm
`gada` library is loaded in all processors with

```
> clusterEvalQ(cl,library(gada))
```

No further setting up is required. After this, when calling `parSBL`, the computing time will automatically decrease depending on the number of processors connected to the cluster.

3.3 Summaryzing results

We use the generic function `summary` to collect all segments for each individual in single object:

```
> allSamples<-summary(myExample)
Warning message:
In summary.parGADA(myExample) :
  All segments are reported. If you want to filter the minimum and maximum
  length of segments, adjust 'length.base'
 (e.g. length.base=c(500,10e6) in base units)
```

This function returns and object of class `summaryParGADA`. The warning message is used to alert the user that all segments will be reported. In some situations, one is only interested in segments with a given size. To do so, the parameter `length.base` should be changed as we illustrate later. Using the generic funtion `print` the following information is obtained

```
> allSamples

-------------------------------------------
Summary results for 8 individuals
-------------------------------------------
NOTE: 814 segments with length not in the range 0-Inf bases and with mean log2ratio
in the range (-0.24,0.14) have been discarded

Number of Total Segments:
 # segments Gains   % Losses    %
        444    38 8.6    406 91.4

Summary of length of segments:
   Min. 1st Qu.  Median    Mean 3rd Qu.    Max.
   2169   20510   58600  221200  167700 8547000

Number of Total Segments by chromosome:
              segments Gains Losses
Chromosome 1        34     2     32
Chromosome 2        23     2     21
Chromosome 3        16     0     16
Chromosome 4        26     0     26
Chromosome 5        16     2     14
Chromosome 6        74     9     65
Chromosome 7        14     2     12
Chromosome 8        29     2     27
Chromosome 9        12     0     12
Chromosome 10       18     5     13
Chromosome 11       23     3     20
Chromosome 12       10     1      9
Chromosome 13        5     0      5
Chromosome 14       15     1     14
Chromosome 15       12     0     12
Chromosome 16       32     2     30
Chromosome 17       25     2     23
Chromosome 18        9     0      9
Chromosome 19       18     2     16
Chromosome 20       11     0     11
Chromosome 21        6     0      6
Chromosome 22       16     3     13
```

If, for instance, we are only interested in altered segments of size between 500 and 10^6 pair of bases, we execute

```
>  allSamples<-summary(myExample, length.base=c(500,10e6))
```

Notice that this function only reports those segments with a mean log2ratio outside the given limits. In this case these limits are (-0.16,0.18) that is assumed to be the interval for segments with 2 copies. By default, these limits are estimated using a threshold approach to classify segments into *Gain* and *Loss* state. The threshold is automatically estimated using the X chromosome of a normal population that includes males (XY) and females (XX). These limits can be changed by the user, by changing the argument `theshold`. As an example

```
> limits<-c(-0.3,0.2)
> allSamples.2<-summary(myExample, length.base=c(500,10e6), threshold=limits)
```

There is a set of functions to simultaneously visualize gains and loses for each individual at both genomic and chromosome level. They are accessed by the generic `plot` function. We can plot information for all individuals in a the same figure using the generic function `plot` and the function `plotWG`. Figure 5 shows

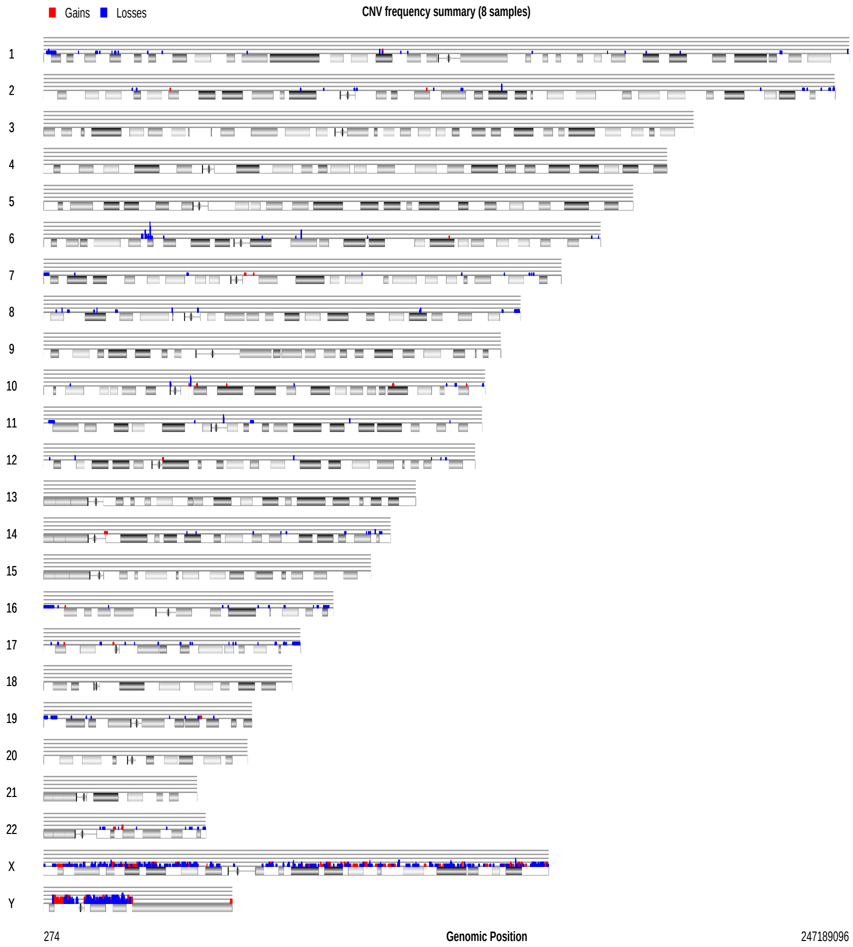

Figure 5: Gains (red colors) and loses (blue colors) relative frequencies for 8 individuals from general population along the entire genomeo

gains and loses corresponding to whole genome analyisis, while Figure 6 shows the same information for chromosome 6. They can be obtained by typing

```
> plotWG(allSamples)
```

and

```
> plot(allSamples,6,show.ind=TRUE)
```

The parameter specifies whether individuals are separated. When a large number of individuals is analyzed it is recommended not to change the default parameter.

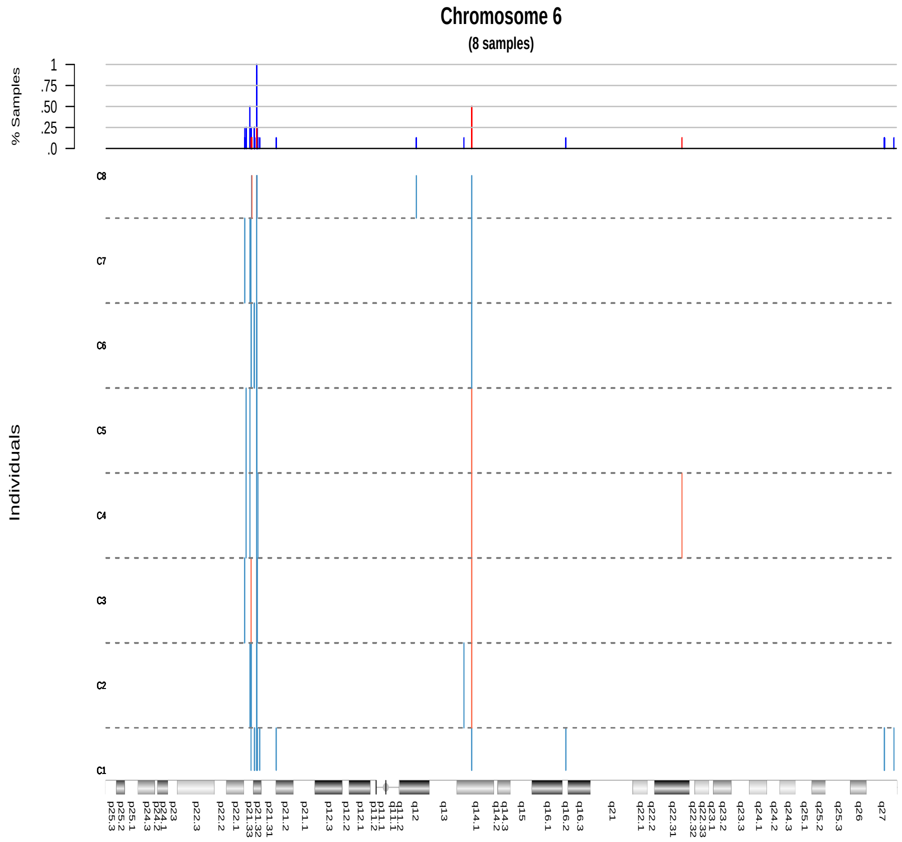

Figure 6: Gains (red colors) and loses (blue colors) for 8 individuals from general population on chromosome 6

The user can also obtain those probes that are altered (gains or losses) in a given proportion of individuals. This can be done using the function `getAlteredProbes` as following

```
> probes<-getAlteredProbes(allSamples, chr=6)
> probes
> probes
$gains
         probe Freq chr      pos
3    rs1064611    2   6 32630503
4    rs1093580    4   6 79056617
6   rs11757159    2   6 32628250
8   rs11759557    2   6 32628011
9   rs11964123    4   6 79052979
14  rs16889854    4   6 79081009
15  rs16889859    4   6 79082584
31  rs28490179    2   6 32626983
37  rs28880026    2   6 32625376
47  rs34182525    2   6 32631416
48  rs34781832    2   6 32628606
49  rs34867789    2   6 32629229
50   rs3819713    2   6 32624385
62   rs6911209    4   6 79065940
65   rs6918807    4   6 79063712
67   rs6931912    4   6 79065999
68   rs6932920    4   6 79059458
69   rs7749022    4   6 79075016
70   rs7773124    4   6 79090197
71   rs7774454    4   6 79077999
72    rs818251    2   6 79031111
73    rs818253    3   6 79031809
74    rs818258    4   6 79034386
75    rs818262    4   6 79036117
76    rs818280    4   6 79088461
77    rs818284    4   6 79083083
78    rs818285    4   6 79083049
79    rs818288    4   6 79078423
80    rs818290    4   6 79077158
81    rs818295    4   6 79069278
82    rs818301    4   6 79056822
83    rs818310    4   6 79042356
84    rs818313    4   6 79039487
94   rs9361392    4   6 79067895
97   rs9443550    4   6 79083326
98   rs9448350    4   6 79069674
99   rs9448356    4   6 79076024
100  rs9448357    4   6 79076473
101  rs9448361    4   6 79086086
103   rs964927    4   6 79070425

$losses
           probe Freq chr      pos
1     cnv30178p1    2   6 30311634
2     cnv30178p3    2   6 30312496
3     cnv30180p1    2   6 30320931
4     cnv30180p2    2   6 30321486
5     cnv30180p4    2   6 30322754
21    cnv30813p1    2   6 32066939
```

```
22    cnv30813p3    2    6 32067243
23    cnv30813p5    2    6 32067459
24   cnv30814p12    2    6 32068749
25   cnv30814p18    2    6 32069461
26    cnv30814p4    2    6 32067857
27    cnv30815p1    2    6 32069619
28   cnv30815p12    2    6 32070703
29   cnv30815p18    2    6 32071423
30    cnv30817p1    2    6 32073734
...
```

Notice that this function requires the argument `chr` corresponding to a desired chromosome. By default, this function only returns those probes that are altered (gains and losses in two different data frames) in more than 10% of samples. This can be changed by using the argument `min.perc`. For example

```
probes2<-getAlteredProbes(allSamples, chr=6, min.perc=0.50)
```

will return the probes that present a gain or a loss in more that 50% of individuals. Another useful tool is the function `exportToBED` to export data

```
> exportToBED(allSamples)
File BED.txt has been generated at /home/jrgonzalez/CREAL/GADA
```

This function generates a file called "BED.txt" that contains the required information to be displayed in most popular genome browsers (UCSC `http://genome.ucsc.edu/`, ENSEMBL `http://www.ensembl.org/index.html`, ...)

4 Multivariate analysis of segmented data

As an illustration of the type of analysis that can follow data segmentation, we show the multivariate discrimination of three HapMap populations (90 CEU, 90 YRI, 45 CBH+ 45 JPT). CEL files for each of the samples are available at http://www.hapmap.org. We used `Aroma.Affimetrix` for its normalization and `GADA` for its segmentation, as described in the previous sections.

We have implemented a set of functions to reduce the data set, without much loss of information, and to perform a multi-class discrimination and variable ranking.

The output of `GADA` for a set of subjects is an object of class `ParGADA` that can be used to build a matrix which encodes the copy number status of each probe and subject in the sample. Performing a segmentation for each separate populations produces a `ParGADA` object for each group, which can be recovered and summarized accordingly

```
#change to CEU directory
ParAffyData <- setupParGADAaffy(log2ratioCol=4,NumCols=4);
Samples.CUE<-summary(ParAffyData,length=c(500,6e9));

#change to YRI directory
ParAffyData <- setupParGADAaffy(log2ratioCol=4,NumCols=4);
Samples.YRI<-summary(ParAffyData,length=c(500,6e9));

#change to CBH-JPT directory
ParAffyData <- setupParGADAaffy(log2ratioCol=4,NumCols=4);
Samples.CJ<-summary(ParAffyData,length=c(500,6e9));
```

The matrix of segment callings, for all probes and subjects, is obtained with the function `getReducedData`. Its arguments are the concatenation of the segmentation results in a single list

```
segments<-c(Samples.CUE,Samples.YRI,Samples.CJ)
```

and the genomic positions of each probe stored in the `gen.info` variable (described above). Both `segments` and `gen.info` for the HapMap samples are readily available at `http://groups.google.com/group/gadaproject`. From these quantities, a reduced matrix is obtained with the command

```
mat.f<-getReducedData(segments, gen.info, varSimil=0.99, subVariation=0.90)
cnv.blocks<-attr(mat.f,"cnv.blocks")
matrixPlot(mat.f)
```

Matrix reduction is controlled with the parameters `varSimil` and `subVariation`. Blocks of neighboring probes that do not differ in more than `varSimil` % across all the subjects in all populations are created as new summary variables. Their values are those of the first probes in each block, detailed in the attribute `attr(mat.f,"cnv.blocks")`. A further reduction is performed with `subVariation`. In this case, blocks that are relatively constant across subjects are discarded as uninformative. Specifically, if `subVariation` is set to 0.9 then only columns taking different values for at least 10% across all samples are kept. The final matrix concatenates the populations into subject blocks and can be displayed by `matrixPlot`. Note that the columns of the matrix are `factors`, consistent with the fact that copy number status (losses, no-change and gains) are categorical variables. Their levels are (-1,0,1). The resulting matrix of the example can be downloaded into the working directory at `http://groups.google.com/group/gadaproject` and recovered into the R session by

```
load("HapMap270reducedData.Rdata")
```

from which the multivariate analysis of the example can be resumed.

We use a discrimination analysis based on multiple correspondence analysis to rank variables, according to the variable's correlation to the axis defined by each population, in the principal component subspace. The discrimination and ranking of the variables follows from

```
cp<-discrimin.cnv(mat.f,pop.cla)
var.rank<-rank.variables(cp,cnv.blocks=cnv.blocks)
```

where `pop.cla` is population labeling for each subject. The entries of this vector and rows of `mat.f` must correspond to the same subject.

The first rows of the the ranking for the HapMap sample illustrate the most relevant blocks of variables in the discrimination of particular populations.

```
> var.rank[1:10,]

            probe num.pr   pos.inf   pos.sup chr correlation population
1   BlkCnv6Chr3.-1     11  46819191  46822621   3       0.816        YRI
2   BlkCnv3Chr3.-1     17  46777922  46794735   3       0.808        YRI
3  BlkCnv42Chr17.1     92  41570665  41707908  17       0.764        CEU
4  BlkCnv43Chr17.1      3  41708649  41711411  17       0.756        CEU
5  BlkCnv44Chr17.1      4  41717787  41719992  17       0.748        CEU
6   BlkCnv2Chr3.-1      5  46776822  46777250   3       0.741        YRI
7  BlkCnv50Chr4.-1      5  69079062  69096368   4       0.710    CBH-JPT
8   BlkCnv5Chr3.-1      1  46807086  46807086   3       0.708        YRI
9  BlkCnv49Chr4.-1     10  69057944  69079058   4       0.693    CBH-JPT
10 BlkCnv48Chr4.-1      1  69057756  69057756   4       0.693    CBH-JPT
```

The variable `BlkCnv6Chr3.-1`, for instance, stands for the binary variable indicating deletions $(.-1)$ in the CNV block number 6 in chromosome 3. The data frame encodes the corresponding number of segments in the block and its genomic inferior and superior positions. As an example, we show the percentage copy number alterations across populations for this particular block

```
     CBH-JPT CEU YRI
  0      100  97  21
  -1       0   3  79
```

Note that a deletion in this block is, indeed, specific to the YRI population. Selecting the first 87 variables, which have correlations higher than 0.5, we finally perform multiple correspondence analysis on them, as an unsupervised classification

```
select<-getNamesProbes(var.proj, min.correlation=0.5)
cm<-dudi.acm(mat.f[select], scan=FALSE, nf=3)
plot(cm, pop.cla,which.axes=c(1,2), var=FALSE, pnt=0.7)
```

Figure 7 shows the classification that it is achieved with only 87 blocks of CNVs for the HapMap sample.

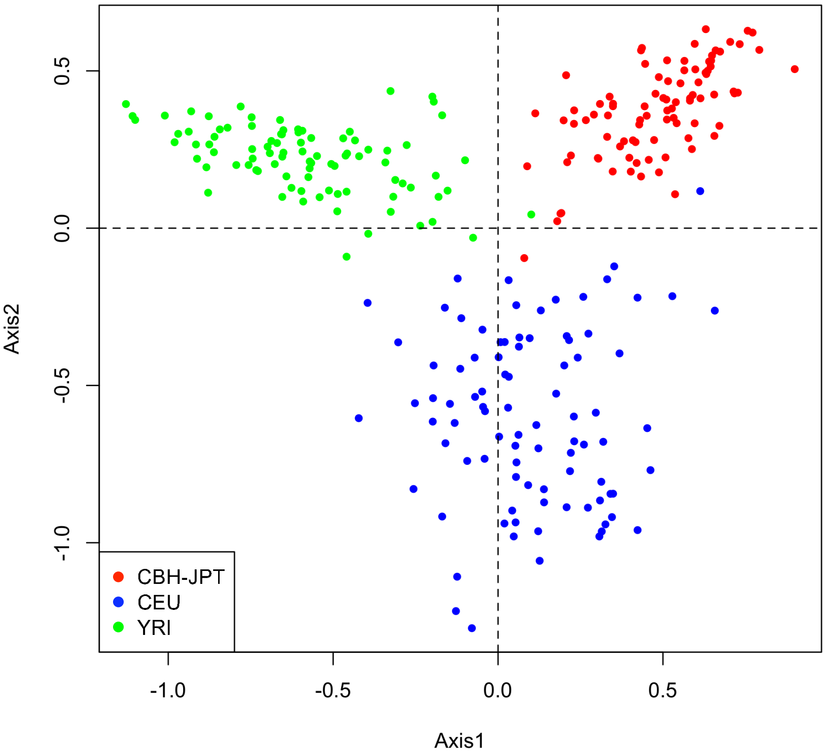

Figure 7: Multiple correspondence analysis for 87 CNV blocks of the HapMap sample. Populations are clearly differentiated with the first two principal axes, that account for 27% of the total variance.

5 Association analysis

Association analysis for multiple CNVs can be performed using `multiCNVassoc` function. We will use a simulated data for which case-control information is generated for HapMap samples. We have randomly generated cases and controls. In order to find some signals, we have generated different proportion of cases for YRI population. Data is available at the google group page `http://groups.google.com/group/gadaproject/web/testing` and can be loaded by typing:

```
load("HapMap270reducedData.Rdata")
```

First, we perform a MCA in order to consider population stratification. We save the first two eigen-vectors to be used in the adjusted models

```
dd<-dudi.acm.fortran(mat.f,scan=FALSE, nf=5)
comp1<-dd$li[,1]
comp2<-dd$li[,3]
```

After that, association (crude analysis) is done by typing

```
ans0<- multiCNVassoc(mat.f, casco~1)
```

We can also consider population stratification in the association analysis by fitting the following models

```
ans<- multiCNVassoc(mat.f, casco~comp1+comp2)
```

A list containing the CNVs associated with cases, sorted by p-values corrected for multiple comparisons using Benjamini-Hockberg's method is obtained as following

```
> getPvalBH(ans0,cnv.blocks)[1:20,]
          region         pval      pval.BH
1  BlkCnv42Chr17 7.063842e-19 1.040504e-15
2  BlkCnv43Chr17 1.716604e-17 1.264279e-14
3  BlkCnv44Chr17 2.896770e-17 1.422314e-14
4  BlkCnv32Chr12 7.061229e-16 2.600298e-13
5  BlkCnv31Chr12 7.421259e-15 2.186303e-12
6  BlkCnv41Chr17 1.455019e-14 3.572072e-12
7  BlkCnv30Chr12 9.261514e-14 1.948887e-11
8  BlkCnv40Chr17 1.039148e-12 1.913331e-10
9  BlkCnv33Chr12 1.187513e-12 1.943564e-10
10 BlkCnv39Chr17 6.314224e-11 9.300852e-09
11  BlkCnv80Chr8 1.052788e-10 1.409779e-08
12  BlkCnv34Chr4 1.230331e-10 1.510231e-08
13  BlkCnv81Chr8 6.391850e-10 7.242458e-08
14  BlkCnv78Chr8 8.311588e-10 8.744977e-08
15  BlkCnv83Chr8 9.031225e-10 8.868663e-08
16   BlkCnv4Chr2 1.542300e-09 1.135904e-07
17   BlkCnv7Chr2 1.342954e-09 1.135904e-07
18  BlkCnv21Chr3 1.523509e-09 1.135904e-07
19  BlkCnv22Chr3 1.523509e-09 1.135904e-07
20  BlkCnv79Chr8 1.407583e-09 1.135904e-07
...
```

Finally, a plot with $-\log_{10}$ p-values can be obtained (see Figure 8) by executing

```
plot(ans0, cnv.blocks, cex=1)
plot(ans, cnv.blocks, cex=1)
```

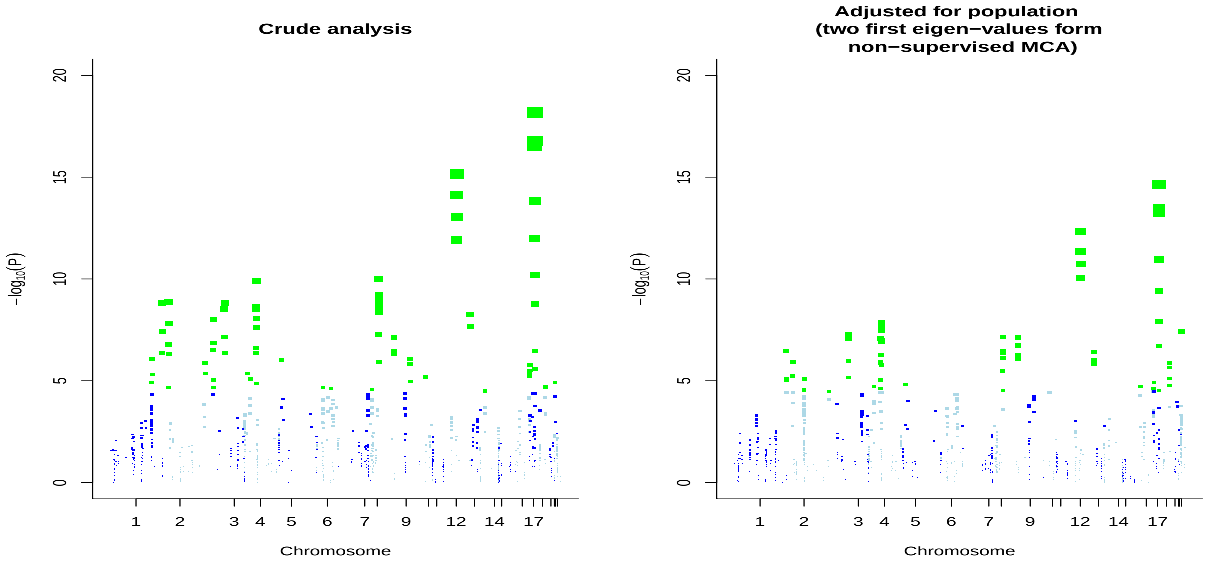

Figure 8: Association analysis for 1,473 blocks for HapMap data using simulated case-control status.

6 Exporting data from Illumina and Affymetrix platforms to gada

6.1 Exporting data from Bead Studio

The BeadStudio tool, which is available at `http://www.illumina.com/`, allows information to be provided in either a unique text file or a file per individual (or groups of individuals). The raw intensities for all individuals in a unique file can be exported shown in Figure 9 and Figure 10. Figure 9 shows how to select the columns of interest, and Figure 10 how this information can be exported.

In order to obtain a different file for each individual, the user has to select “Final Report” as illustrated in Figure 11. Then, he must select 1 in the file “Samples/File” (Figure 12). Only “Log R Ratio” is necessary to be displayed, but other fields such as “B Allele Freq” can be exported to analyze the data using other programs.

The resulting files for each subject have the following format, if the user have selected genotype, log2ratio, and B allele frequency

```
Name Chr Position GType Log R Ratio B Allele Freq
rs10000010 4 21227772 BB -0.1157656 0.9982474
rs10000023 4 95952929 AB -0.1266638 0.4817977
rs10000030 4 103593179 AB 0.0514016 0.5103833
rs1000007 2 237416793 AB 0.138847 0.4689891
rs10000092 4 21504615 AA 0.01165604 0.00370151
rs10000121 4 157793485 BB -0.02247738 0.9751745
rs1000014 16 24325037 BB 0.0001281789 0.9989412
rs10000141 4 33810744 BB -0.01945104 0.9805357
rs1000016 2 235355721 AA -0.2437027 0.00094727
rs10000169 4 77575270 AA 0.08803905 0
rs1000022 13 99259220 AA -0.2494328 0
rs10000272 4 189927377 AA -0.1513728 0.002721502
...
```

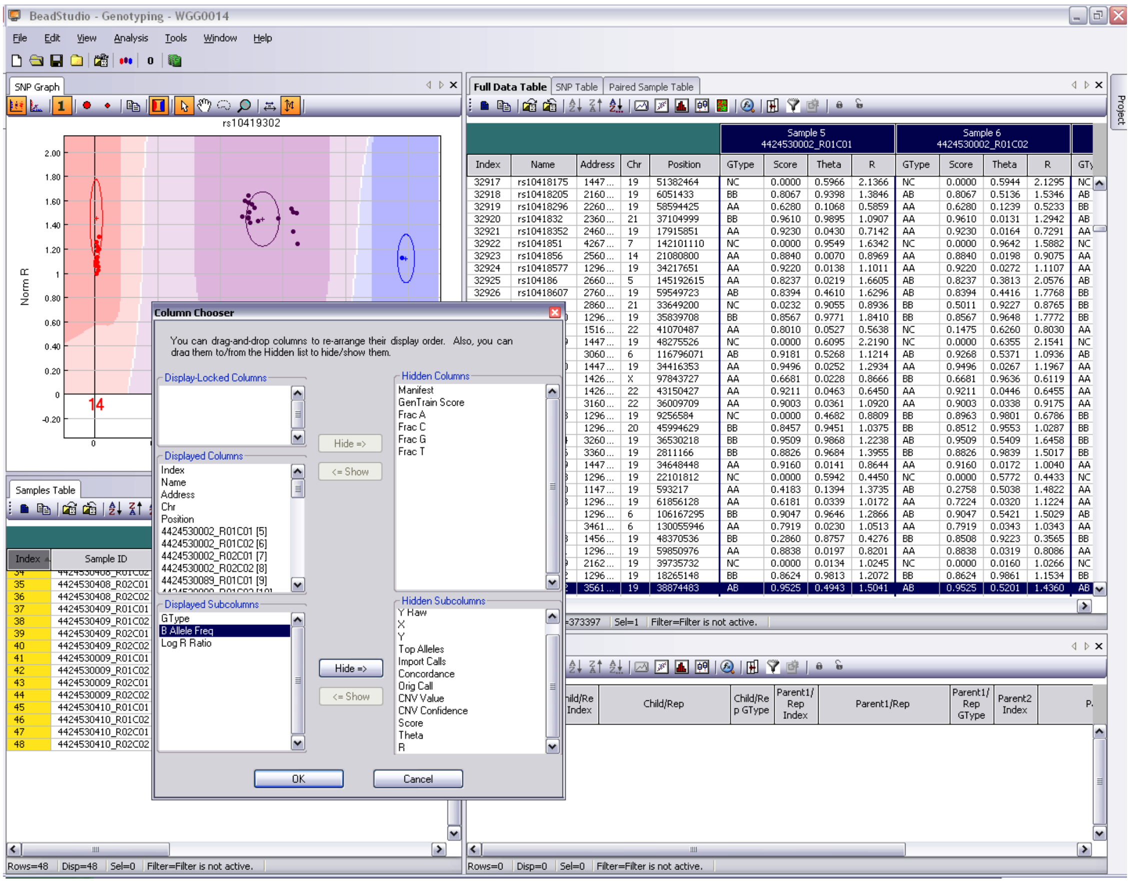

Figure 9: Exporting log2ratios from BeadStudio tool in a unique file

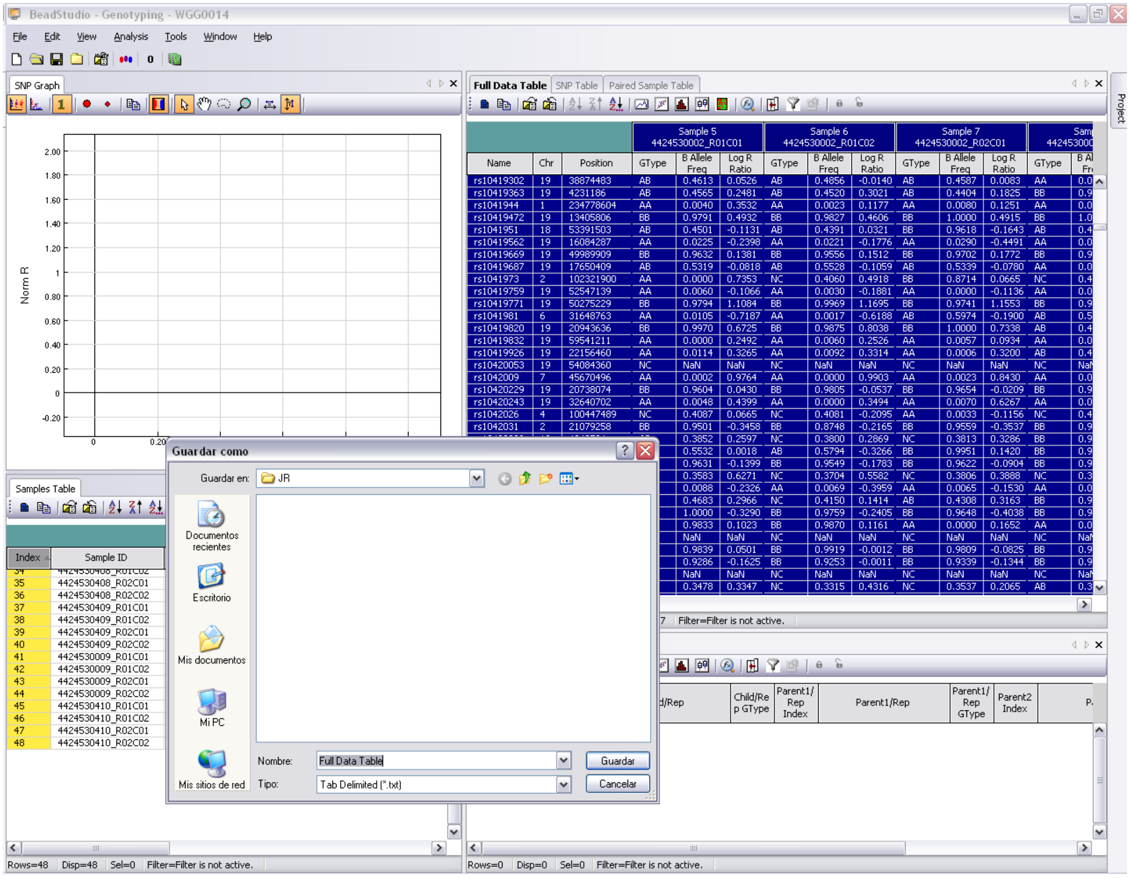

Figure 10: Exporting log2ratios from BeadStudio tool in a unique file

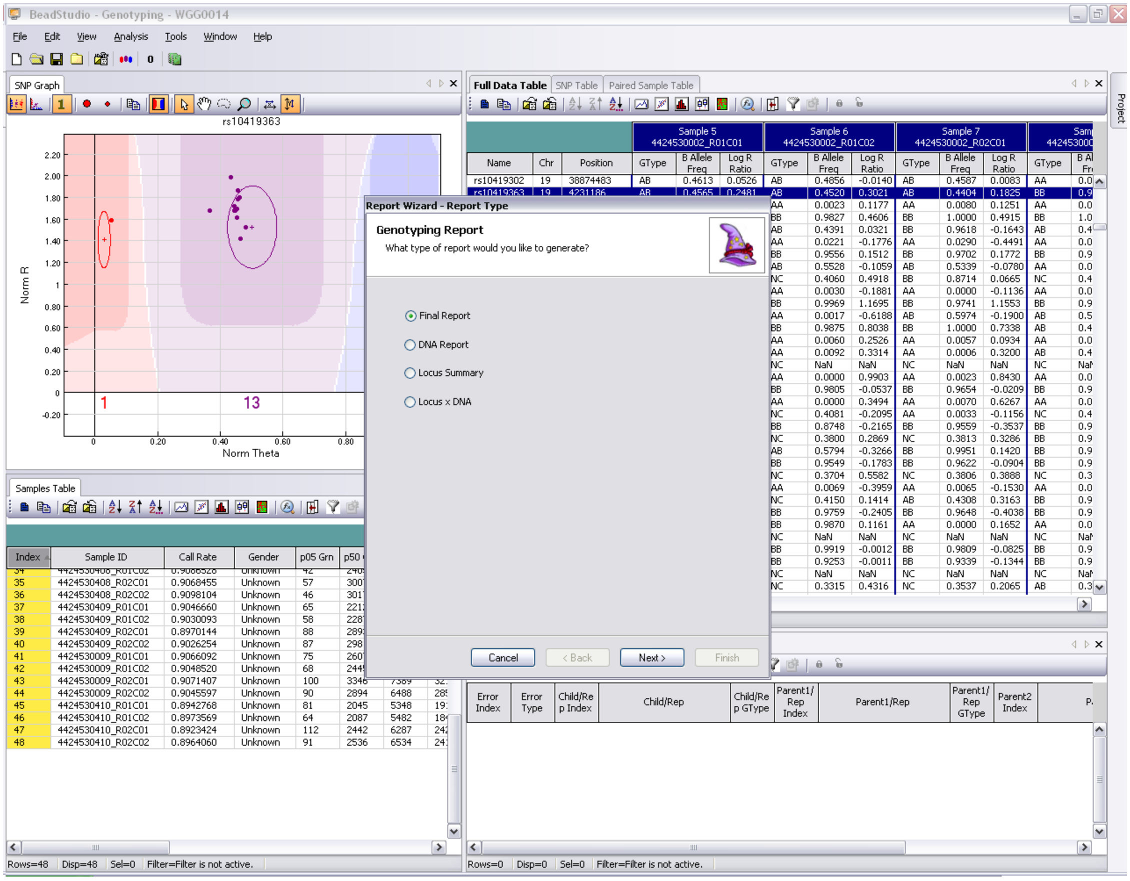

Figure 11: Exporting log2ratios from BeadStudio tool in different files

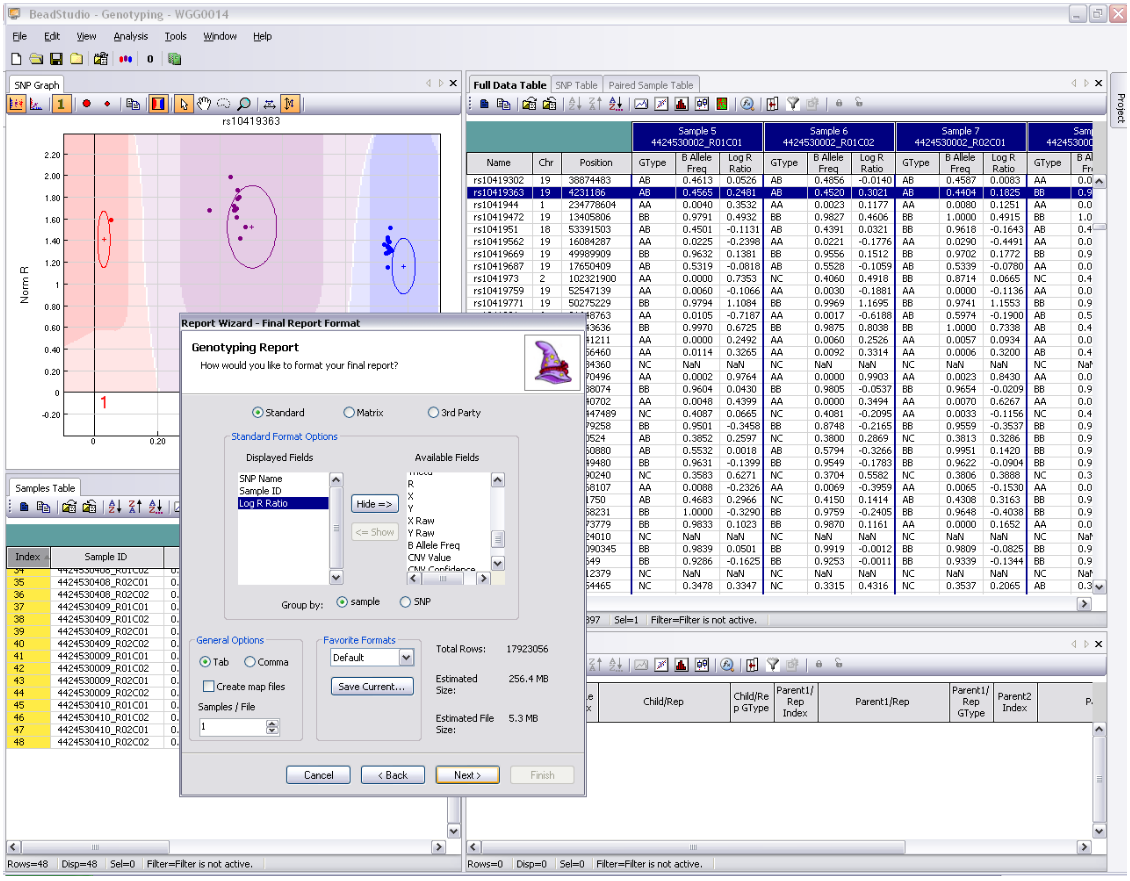

Figure 12: Exporting log2ratios from BeadStudio tool in different files

6.2 Exporting data from Affymetrix genotyping console (GTC)

The Affymetrix Genotyping Console 3.0 (GTC3), which can be downloaded from `http://www.affymetrix.com/products_services/software`, can also be used to extract normalized log2ratio intensities from a collection of CEL files. After analyzing the data with the GTC3 copy number tool, the raw instensities are exported by selecting "Export Copy Number/ LOH Results" with the right button (Figure 13).

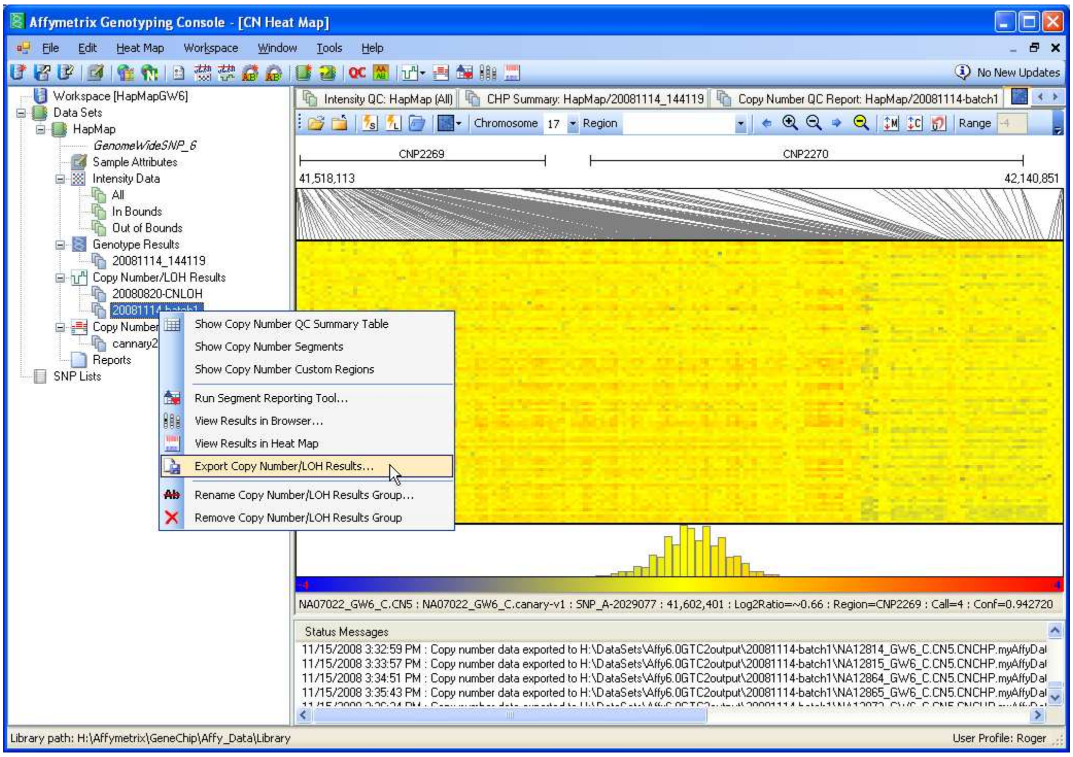

Figure 13: Exporting log2ratios from the Affymetrix genotyping console 3.0 (GTC3)

Only the log2ratio intensities are necessary to be exported (Figure 14). Once exported, the resulting files containing the data can be found on the output folder that was specified on the Copy Number Tool (Figure 14).

The resulting files for each file should have the following format:

```
#comments
#comments
...
#comments
ProbeSet        Chromosome      Position        Log2Ratio
CN_473963       1       51586   -0.257667
CN_473964       1       51659   -0.264712
CN_473965       1       51674   -0.0436751
CN_473981       1       52771   -0.40294
CN_473982       1       52788   0.134605
CN_497981       1       62627   -0.0063667
CN_502615       1       75787   -0.677508
CN_502613       1       75849   -0.343111
```

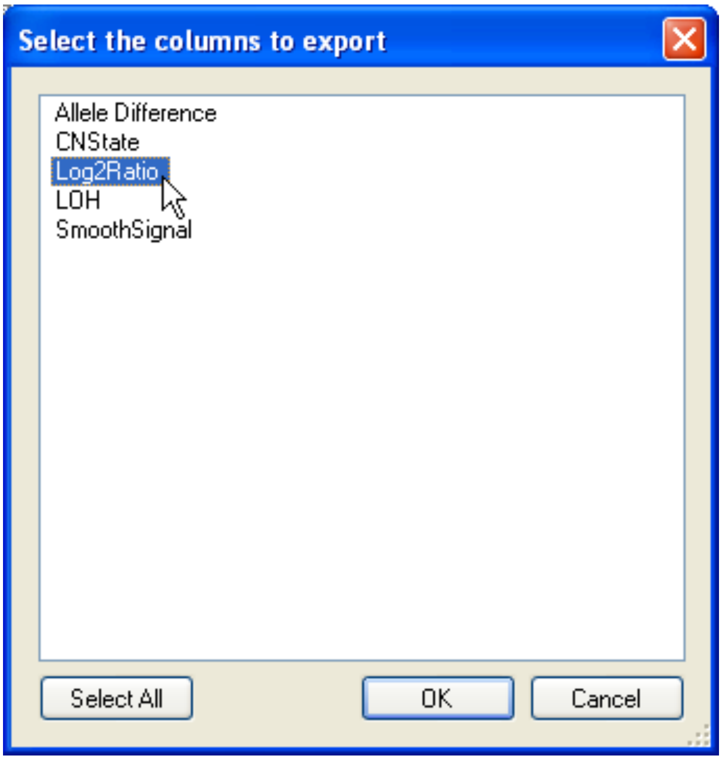

Figure 14: Exporting log2ratios from the Affymetrix genotyping console 3.0 (GTC3)

```
CN_502614       1       76175   -1.44067
...
```

6.3 Exporting data from Affymetrix power tools (APT)

Alternativelly the log2ratio intensities can be extracted with the Affymetrix power tools (APT) available from `http://www.affymetrix.compartners_programs/programs/developer/tools/powertools.affx`. This tools provide more flexibility on the normalization procedures and settings that can be used:

```
$ apt-copynumber-workflow \
    --adapter-type-normalization true \
    --reference-output results-dir/MySamplesReference.a5.ref \
    --set-analysis-name MySamples \
    --cdf-file GenomeWideSNP_6.cdf \
    --chrX-probes GenomeWideSNP_6.chrXprobes \
    --chrY-probes GenomeWideSNP_6.chrYprobes \
    --special-snps GenomeWideSNP_6.specialSNPs \
    --netaffx-snp-annotation-file GenomeWideSNP_6.na25.annot.csv \
    --netaffx-cn-annotation-file GenomeWideSNP_6.cn.na25.annot.csv \
    --delete-files true \
    --o results_dir \
    --text-output true \
    --delete-files false \
    --cel-files *.CEL
```

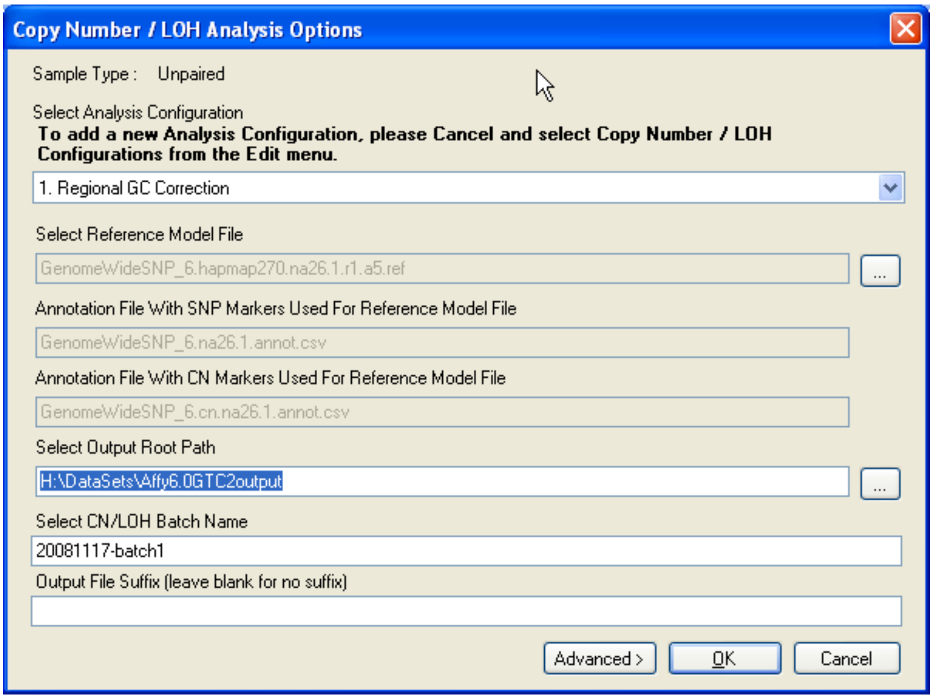

Figure 15: Copy number tool dialog box that specifies the results folder

The APT user manual provide more detailed explanation on all the different possible settings. We use `--text-output` option to produce the text files with the following `GADA` format:

```
$ head -500 NA06985_GW6_C.MyTest.CN5.CNCHP.txt

#Comments
....
#Comments
#Comments
ProbeSetName    Chromosome      Position        CNState Log2Ratio       SmoothSignal    LOH     Allele Difference
CN_473963       1       51586   2       -0.257667       1.054558        nan     nan
CN_473964       1       51659   2       -0.264712       1.054389        nan     nan
CN_473965       1       51674   2       -0.043675       1.054354        nan     nan
CN_473981       1       52771   2       -0.402939       1.051817        nan     nan
CN_473982       1       52788   2       0.134605        1.051777        nan     nan
CN_497981       1       62627   2       -0.006367       1.029375        nan     nan
CN_502615       1       75787   2       -0.677508       1.000571        nan     nan
CN_502613       1       75849   2       -0.343111       1.000438        nan     nan
CN_502614       1       76175   0       -1.440673       0.999744        nan     nan
CN_502616       1       76192   0       -2.477916       0.999708        nan     nan
CN_502843       1       88453   2       -0.135097       0.974336        nan     nan
CN_466171       1       218557  2       -0.030157       1.597003        nan     nan
CN_468414       1       218926  2       -0.475484       1.597018        nan     nan
CN_468412       1       219009  2       -0.045742       1.597021        nan     nan
CN_468413       1       219024  2       -0.050614       1.597022        nan     nan
...
```

7 Tutorial session with Affymetrix data

7.1 Analyzing a single Affymetrix array

The data used in this example can be downloaded from:

```
> download.file("http://www.creal.cat/jrgonzalez/GADA/NA12248_GW6_C.MyTest.CN5.CNCHP.txt",
+               "./NA12248_GW6_C.MyTest.CN5.CNCHP.txt")
trying URL 'http://www.creal.cat/jrgonzalez/GADA/NA12248_GW6_C.MyTest.CN5.CNCHP.txt'
Content type 'text/plain' length 97542566 bytes (93.0 Mb)
opened URL
==================================================
downloaded 93.0 Mb
```

A single Affymetrix array can be imported to gada by executing:

```
> dataAffy <- setupGADAaffy("NA12248_GW6_C.MyTest.CN5.CNCHP.txt",NumCols=8,log2ratioCol=5)
Read 14507536 items
```

We use NumCols=8, log2ratioCol=5 for the results exported from APT tools (Section 6.3), or we should NumCols=4 and log2ratioCol=4 for Affymetrix Genotyping Console, Section 6.2.

We check data import entering

```
> dataAffy
Object of class 'setupGADA' (Affy data)
-------------------------------------------
  Number of probes: 1813441 (0 missing values)

  Number of probes by chromosome:
     1      2      3      4      5      6      7      8      9     10     11
141348 148812 123956 116379 112136 109149  97441  95116  79106  90328  86362
    12     13     14     15     16     17     18     19     20     21     22
 84371  64071  55219  51570  52002  44888  50461  29067  41816  24208  23172
     X      Y
 84315   8148
```

We can also visualize the raw data in a plot like in Figure 16 using

```
> plotRatio(dataAffy, num.points=50000)
```

The same information can be detailed as in Figure 17 for chromosome 12

```
> plotRatio(dataAffy, chr=12, num.points=50000)
```

The segments are obtained by the two step approach consisting of the SBL and BackwardElimination procedures, as described in section 2.3.

```
> step1<-SBL(dataAffy, aAlpha=0.5, estim.sigma2=TRUE)
    The estimated sigma2 = 0.02658385
> step1
Sparse Bayesian Learning (SBL) algorithm
sigma2 = 0.0266
--------------------------------------------------------------
   chromosome discontinuities numit    tolerance
1           1            3011  1087 9.941786e-09
2           2            3168   897 9.971203e-09
3           3            2439   690 9.890185e-09
4           4            2458  2630 9.973668e-09
5           5            2213  1697 9.998423e-09
6           6            2134   591 9.958981e-09
7           7            2080  1270 9.936370e-09
8           8            1913  1489 9.976727e-09
9           9            1651  3488 9.969362e-09
10         10            1926   734 8.913446e-09
```

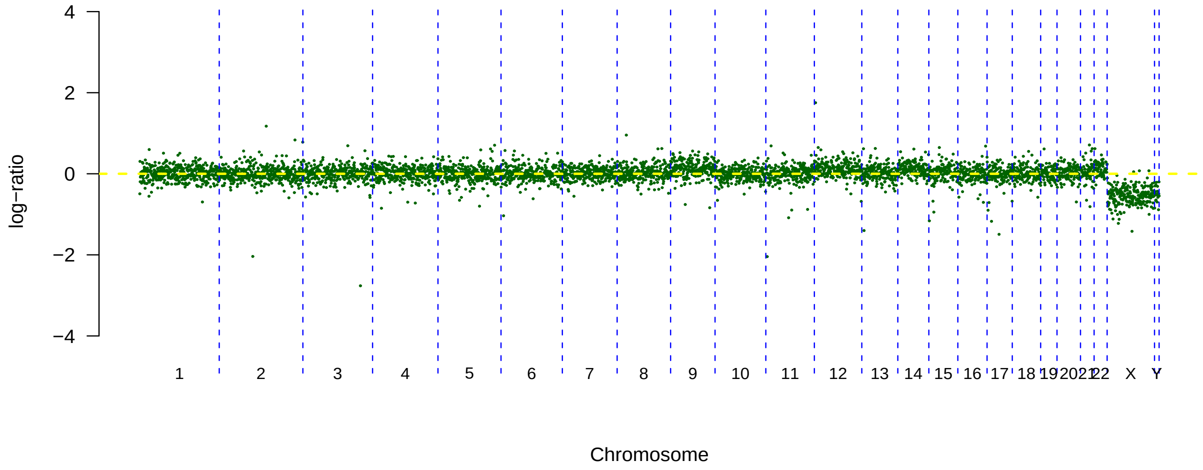

Figure 16: Affymetrix log-ratio intensities by chromosome

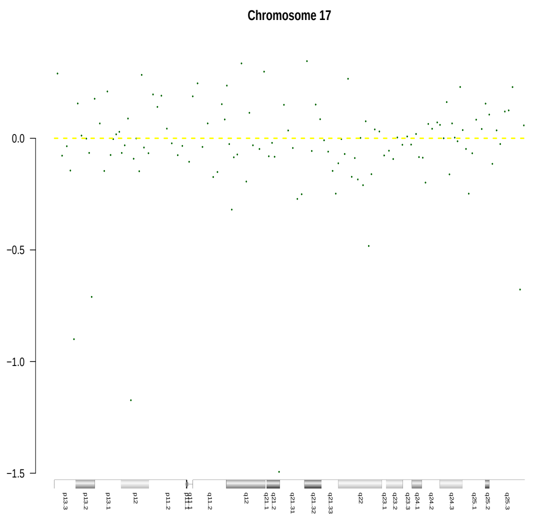

Figure 17: Affymetrix log-ratio intensities for chromosome 17

```
11         11           1700   661 9.812538e-09
12         12           1733   520 9.690517e-09
13         13           1385   564 9.858486e-09
14         14           1271   682 9.960733e-09
15         15           1069   433 9.733707e-09
16         16           1156   618 9.905047e-09
17         17           1026   457 9.924282e-09
18         18            991   680 9.499992e-09
19         19            720   327 6.731085e-09
20         20            843   413 9.827422e-09
21         21            555   360 9.616085e-09
22         22            600   371 9.603308e-09
23          X           4016  2775 9.506493e-09
24          Y            183   608 3.030268e-09
```

```
> step2<-BackwardElimination(step1,T=6,MinSegLen=3)
> step2
Sparse Bayesian Learning (SBL) algorithm
SBL and Backward Elimination with T=6 and minimum length size=3
sigma2 = 0.0266
-------------------------------------------------------------
   chromosome discontinuities
1           1              55
2           2              69
3           3              28
4           4              50
5           5              37
6           6              27
7           7              45
8           8              36
9           9              13
10         10              23
11         11              27
12         12              20
13         13              25
14         14              21
15         15              22
16         16              16
17         17              18
18         18              14
19         19               6
20         20              16
21         21               5
22         22              15
23          X              60
24          Y               4
```

The advantage of using a two step approach is that we can flexibly adjust T, remove or add breakpoints that will follow in significance, without fitting the entire SBL model again.

```
> summary(step2)
---------------------------------------
Sparse Bayesian Learning (SBL) algorithm
Backward Elimination procedure with T=6 and minimum length size=3
 Number of segments =  676
 Base Amplitude of copy number 2: chr 1:22:-0.0024, X=-0.5423, Y=-0.5282
 Gains (1) and Loses (-1) with respect Base Amplitude
---------------------------------------
```

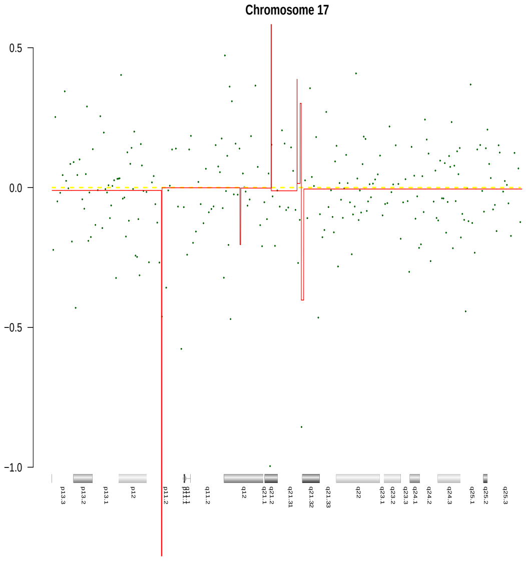

Figure 18: Affymetrix log-ratio intensities and segments for chromosome 17

```
     IniProbe  EndProbe LenProbe      MeanAmp chromosome State
1       51586     76192       10 -0.408413323          1    -1
3     1617766   1640775       11 -1.104301596          1    -1
4     1642243   1662451        7 -1.967756180          1    -1
6     5146486   5147215        3 -0.696282657          1    -1
7     5150357  17063437     6590 -0.014075678          1    -1
8    17076072  17131709       49  0.191480187          1     1
10   25468522  25519264       21 -0.259745800          1    -1
12   40794663  40800797        4 -0.629174323          1    -1
14   72528689  72541492       11 -0.474053142          1    -1
15   72541512  72547710        8  2.256061427          1     1
16   72551656  72569602       17  1.231081324          1     1
17   72569988  72575080        4  1.812702177          1     1
19   72578384  72581327        3  1.707572010          1     1
20   72581344  72582418        4  0.787632927          1     1
21   72583514  72583724        4  2.123788677          1     1
23  105820716 105825648       21  0.346537724          1     1

...

666 143439162 143445353        3 -1.216180667          X    -1
668 147536256 147554906       14 -0.844484429          X    -1
670 153177486 154582680      634 -0.498188104          X     1
671 154616633 154887040       42  0.015083500          X     1
673   4613756   4665080        3 -1.142382667          Y    -1
675   5584359   5620349        4 -1.075439750          Y    -1
```

The visual representation of the recovered segments can be obtained by

```
> plotRatio(step2, chr=17)
```

7.2 Analyzing a collection of 90 Affymetrix arrays

Affymetrix data for 60 CEU samples are available at `http://www.creal.cat/jrgonzalez/GADA/20081114-AffyGTC301.rar`. We begin downloading all the `*.txt` files (exported GTC3 or APT) in the local `./rawData/` folder. No other other file should be placed here since all files ending with `*.txt` in that folder will be imported;

```
> ParAffyData <- setupParGADAaffy(log2ratioCol=4,NumCols=4);

Creating objects of class setupGADA for all input files...
  Applying setupGADAaffy for 90 samples ...
  Importing array:  NA06985_GW6_C.CN5.CNCHP.myAffyData.txt ... Read 7253768 items
   Array # 1 ...done
  Importing array:  NA06991_GW6_C.CN5.CNCHP.myAffyData.txt ... Read 7253768 items
   Array # 2 ...done
...
  Importing array:  NA12892_GW6_C.CN5.CNCHP.myAffyData.txt ... Read 7253768 items
   Array # 90 ...done
Creating objects of class setupGADA for all input files... done
```

Remember to modify `log2ratioCol` and `NumCols` if another format is used.

Once we have imported the data, we can follow exactly the same steps as in the Illumina case in Section 3. We can store the object with the path structure for future analysis, to avoid importing data again.

```
> ## Storing object with the imported data.
> save(ParAffyData,file='ParAffyData.rData');
>
> load("ParAffyData.rData")
>
```

Individual arrays and chromosomes are easily accessed for visualization

```
> ## plot ratio intensities for sample #4
> plotRatio(ParAffyData,Sample=4,num.points=5000)
>
> ## plot ratio intensities for sample #4 and chromosome 2
> plotRatio(ParAffyData,Sample=4,chr=2,num.points=5000)
>
```

The segmetnation analysis can be run as a batch for all samples, or in parallel if we have the `snow` and `Rmpi` packages installed.

```
> ## ## Segmentation for all samples
> parSBL(ParAffyData,aAlpha=0.5,estim.sigma2=TRUE);
Creating SBL directory ...done
Retrieving annotation data ...done
Segmentation procedure for 90 samples ...
   Array # 1 ...    The estimated sigma2 = 0.02662487
   Array # 1 ...done
   Array # 2 ...    The estimated sigma2 = 0.03188311
   Array # 2 ...done
...
   Array # 90 ...    The estimated sigma2 = 0.02679275
   Array # 90 ...done
Segmentation procedure for 90 samples ...done
Warning messages:
1: In FUN(1:24[[24L]], ...) :
  SBL algorithm did not converge after  10000 iterations and change 3.82616197214247e-06
2: In FUN(1:24[[24L]], ...) :
  SBL algorithm did not converge after  10000 iterations and change 7.17238078706828e-07
3: In FUN(1:24[[24L]], ...) :
```

```
  SBL algorithm did not converge after  10000 iterations and change 1.11067555152999e-08
>
```

After the SBL we continue with the BE step using `parBE`

```
> parBE(ParAffyData,T=6,MinSegLen=8)
Retrieving annotation data ...done
Backward elimination procedure for 90 samples ...
   Array # 1 ... ---------------------------------------
Sparse Bayesian Learning (SBL) algorithm
Backward Elimination procedure with T=6 and minimun length size=8
 Number of segments =  516
 Base Amplitude of copy number 2: chr 1:22:-0.0027, X=0.0343, Y=-2.2033
   Array # 2 ... ---------------------------------------
Sparse Bayesian Learning (SBL) algorithm
Backward Elimination procedure with T=6 and minimun length size=8
 Number of segments =  419
 Base Amplitude of copy number 2: chr 1:22:0.0031, X=0.014, Y=-2.1485

...

   Array # 90 ... ---------------------------------------
Sparse Bayesian Learning (SBL) algorithm
Backward Elimination procedure with T=6 and minimun length size=8
 Number of segments =  451
 Base Amplitude of copy number 2: chr 1:22:-2e-04, X=0.0212, Y=-1.9905
Backward elimination procedure for 90 samples ...done
```

The result of the segmentation of all the samples can be summarized by

```
> allSamples<-summary(ParAffyData,length=c(500,6e9));
>
> print(allSamples)
-------------------------------------------
Summary results for 90 individuals
-------------------------------------------
NOTE: 2561 segments with length not in the range 500-6e+09 bases
and with mean log2ratio in the range (-0.28,0.16) have been discarded

Number of Total Segments:
 # segments Gains    % Losses    %
       7913  2305 29.1   5608 70.9

Summary of length of segments:
    Min.  1st Qu.   Median     Mean  3rd Qu.     Max.
     533     5783    15050    67630    48990 21410000

Number of Total Segments by chromosome:
              segments Gains Losses
Chromosome 1       939   287    652
Chromosome 2       716   199    517
Chromosome 3       557   184    373
Chromosome 4       666   148    518
Chromosome 5       401    79    322
Chromosome 6       399    89    310
Chromosome 7       474   159    315
Chromosome 8       482   100    382
Chromosome 9       203    43    160
Chromosome 10      225    77    148
```

```
Chromosome 11       288    84    204
Chromosome 12       334    90    244
Chromosome 13       216    52    164
Chromosome 14       466   151    315
Chromosome 15       304    67    237
Chromosome 16       213    59    154
Chromosome 17       259   121    138
Chromosome 18       148    16    132
Chromosome 19       192    81    111
Chromosome 20       147    98     49
Chromosome 21        30    10     20
Chromosome 22       254   111    143
```

we can adjust the number of breakpoints by modifying the parameter `T` very quickly. If we increase T to 12 for example, the number of detected breakpoints is reduced maintaining only those that are more likely to be true breakpoints.

```
> parBE(ParAffyData,T=12,MinSegLen=10)
...
> allSamples<-summary(ParAffyData,length=c(500,6e9));
> print(allSamples)

-------------------------------------------
Summary results for 90 individuals
-------------------------------------------
NOTE: 384 segments with length not in the range 500-6e+09 bases
and with mean log2ratio in the range (-0.28,0.16) have been discarded

Number of Total Segments:
 # segments Gains    % Losses    %
       3308   866 26.2   2442 73.8

Summary of length of segments:
    Min.  1st Qu.   Median     Mean  3rd Qu.     Max.
     618     7434    27930   114200   101700 16320000

Number of Total Segments by chromosome:
              segments Gains Losses
Chromosome 1       397    87    310
Chromosome 2       305    64    241
Chromosome 3       328   111    217
Chromosome 4       304    78    226
Chromosome 5       144    45     99
Chromosome 6       191    57    134
Chromosome 7       208    66    142
Chromosome 8       207    15    192
Chromosome 9        70     4     66
Chromosome 10       73    22     51
Chromosome 11       88     8     80
Chromosome 12      150    30    120
Chromosome 13       46     4     42
Chromosome 14      156    41    115
Chromosome 15      125    30     95
Chromosome 16       85    21     64
Chromosome 17      156    73     83
Chromosome 18       43     5     38
Chromosome 19       46    11     35
Chromosome 20       75    51     24
```

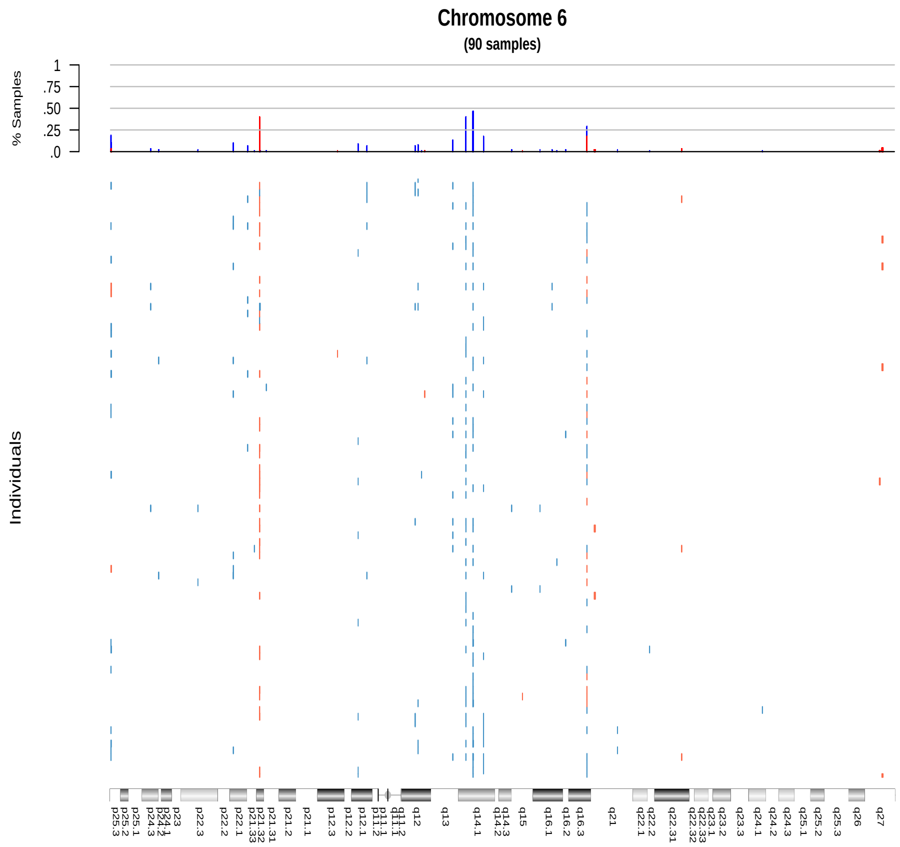

Figure 19: Gains (red) and losses (blue) for 90 CEU individuals on chromosome 6

```
Chromosome 21       10     3      7
Chromosome 22      101    40     61
>
```

An increase of T will reduce the sensitivity to detect true breakpoints, although the false discovery rate (FDR) will also be smaller.

We can plot the information that summarizes all the CNA findings using the functions `plot` and `plotWG`. Figure 5 shows gains and losses across the whole genome, while Figure 6 details the findings for chromosome 6.

```
> plotWG(allSamples)
```

```
> plot(allSamples,6,show.ind=TRUE)
```

The probes that fall on areas containing CNA on chromosome 17 can also be obtained using

```
> altProbes<-getAlteredProbes(allSamples,chr=17)
> altProbes
$gains
            probe Freq chr      pos
6       CN_146278   36  17 41523026
7       CN_146297   62  17 41581088
```

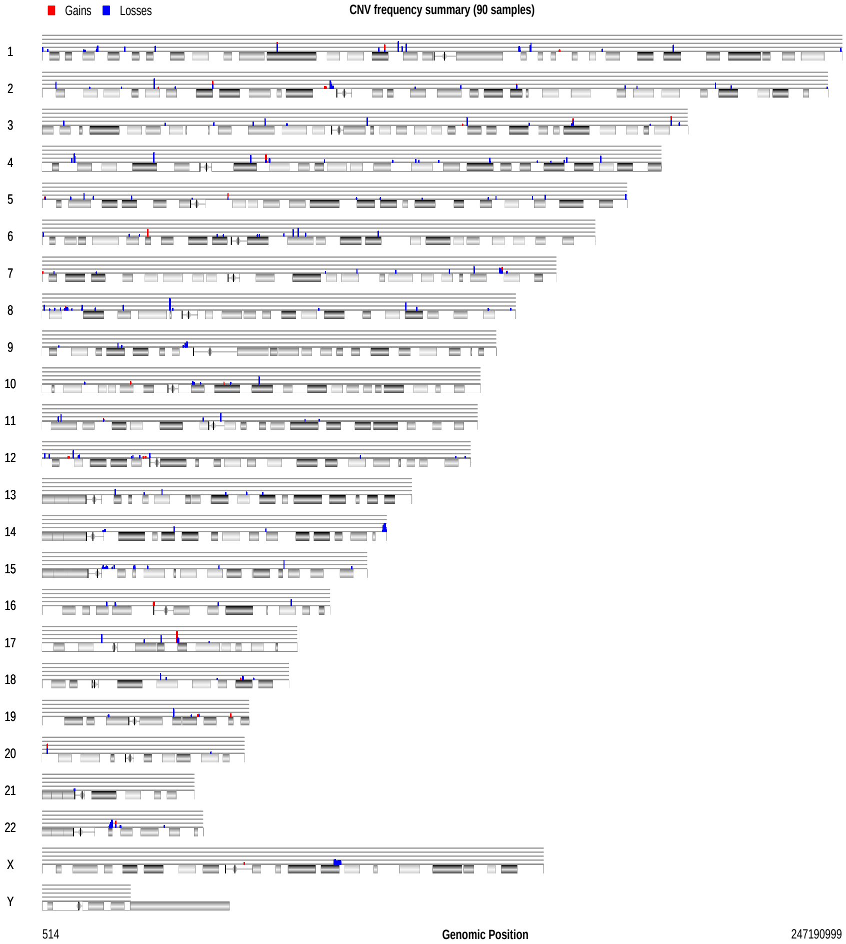

Figure 20: Gains (red) and losses (blue) frequencies for 90 CEU individuals along the entire genome

```
8       CN_146310   62  17 41623467
9       CN_146322   62  17 41667651
10      CN_146343   27  17 41750175


...

243 SNP_A-8534896   62  17 41581663
246 SNP_A-8658169   35  17 41522088

$losses
            probe Freq chr      pos
8       CN_146343   16  17 41750175
12      CN_429094   18  17 41750177
15      CN_739260   18  17 41750183
16      CN_739262   21  17 41756820
17      CN_739264   21  17 41764411

...

91      CN_751784   17  17 18387392
92      CN_751786   15  17 18394150
185 SNP_A-4288097   17  17 18308103
199 SNP_A-8505450   21  17 41927619
```

Finally, the function `exportToBED` can be used to save the CNA segments in BED format

```
> exportToBED(allSamples)
File BED.txt has been generated at /data/cluster1/rpique/datasets/aptDataNew/AffyCeuGW6GTC301
```

which are stored on the BED.txt file,

```
$ head BED.txt
chr1    72541512        72583724        NA06985     300     +       72541512        72583724        255,0,0
chr1    147303136       147438362       NA06985     300     +       147303136       147438362       255,0,0
chr1    147442911       147496455       NA06985     300     +       147442911       147496455       255,0,0
chr1    147509275       147521544       NA06985     300     +       147509275       147521544       255,0,0
chr1    147526028       147703454       NA06985     300     +       147526028       147703454       255,0,0
chr1    246815805       246877269       NA06985     300     +       246815805       246877269       255,0,0
chr1    72541512        72583709        NA06991     300     +       72541512        72583709        0,255,255
chr1    105820716       105823886       NA06991     300     +       105820716       105823886       255,0,0
chr1    110027431       110044464       NA06991     300     +       110027431       110044464       0,255,255
chr1    111179076       111189737       NA06991     300     +       111179076       111189737       255,0,0
...
```

then we can visualize this file on the UCSC genome browser `http://genome.ucsc.edu/` (Figure 21)

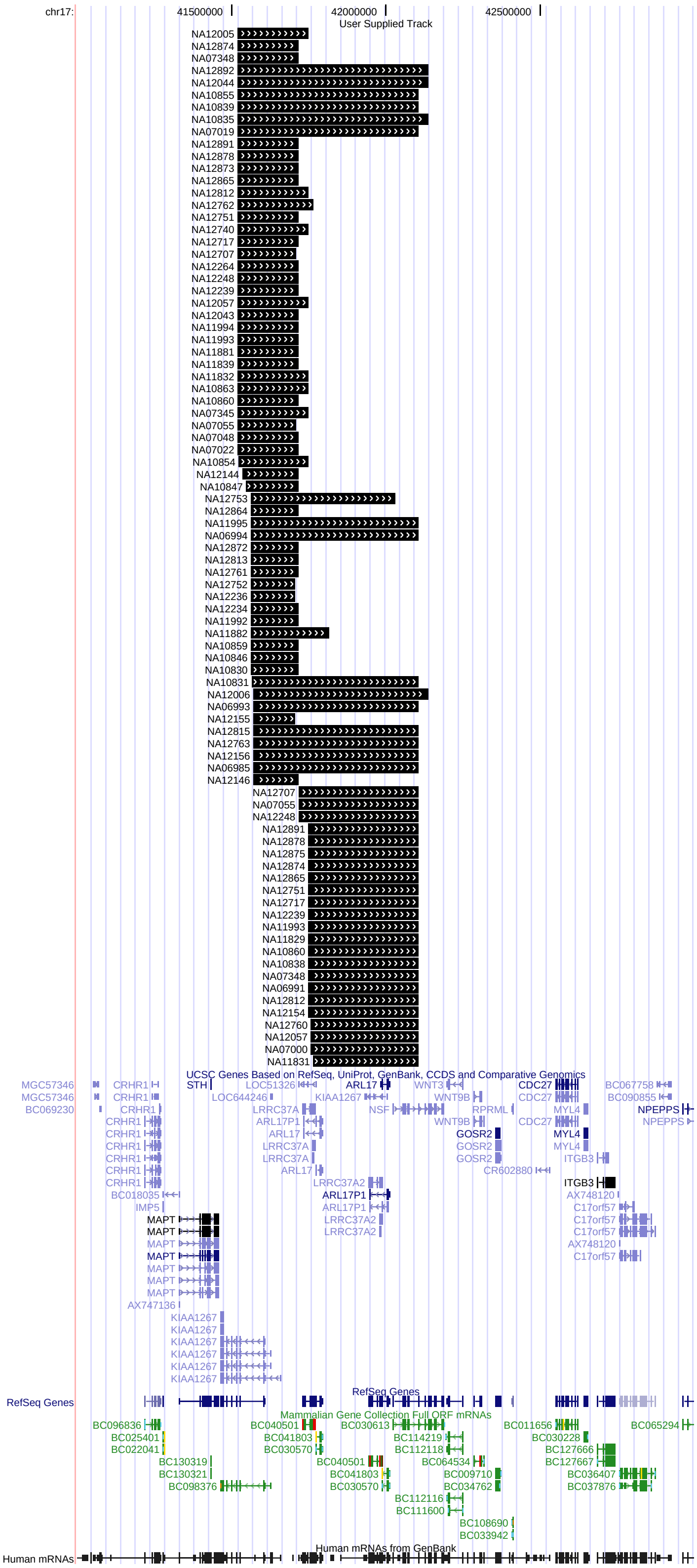

Figure 21: Results on the UCSC browser depicting a known CNV region

8 Connection with Aroma.Affymetrix

GADA can also be called from within `Aroma.Affymetrix` package (`http://groups.google.com/group/aroma-affymetrix/`) which provides a normalization model described in [1] as well as an analysis framework which includes copy number detection and visualization. In this environment, `GADA` segmentation tools are coupled with `Aroma.Affymetrix` package pipeline. In the vignette in `http://groups.google.com/group/aroma-affymetrix/web/total-copy-number-analysis-6-0` GADA is performed by using `GadaModel()` instead of `CbsModel()`.

Following the vignette, we have the set of instructions

```
> library(aroma.affymetrix)
> cdf <- AffymetrixCdfFile$fromChipType("GenomeWideSNP_6", tags="Full") # Specify library files
> cs <- AffymetrixCelSet$fromName("MeduloWithControls", cdf=cdf) # Defining folder with CEL files
> acc <- AllelicCrosstalkCalibration(cs) # Set allelic crosstalk model
> csC <- process(acc, verbose=verbose) # Fit and correct allelic crosstalk
> plm <- AvgCnPlm(csC, mergeStrands=TRUE, combineAlleles=TRUE, shift=+300) #Summarization model
> fit(plm, verbose=verbose) # Fit summarization model
> ces <- getChipEffectSet(plm)
> fln <- FragmentLengthNormalization(ces) #   PCR fragment length normalization (FLN)
> cesN <- process(fln, verbose=verbose)
```

Once the normalization model is normalized and calibrated, the following function adds the `GADAmodel` methods to `Aroma.Affymetrix`:

```
> library(gada)
> addGadaToAromaAffymetrix() # Adds the gadaModel to aroma.affymetrix
```

We can create a `GADAmodel` using:

```
> gada <- GadaModel(cesN,aAlpha=0.8,T=6,MinSegLen=3); #Without reference
> print(gada)
GadaModel:
Name: MeduloWithControls
Tags: ACC,ra,-XY,AVG,+300,A+B,FLN,-XY,a0.8
Chip type (virtual): GenomeWideSNP_6
Path: gadaData/MeduloWithControls,ACC,ra,-XY,AVG,+300,A+B,FLN,-XY,a0.8/GenomeWideSNP_6
Number of chip types: 1
Chip-effect set & reference file pairs:
Chip type #1 of 1 ('GenomeWideSNP_6'):
Chip-effect set:
CnChipEffectSet:
Name: MeduloWithControls
Tags: ACC,ra,-XY,AVG,+300,A+B,FLN,-XY
Path: plmData/MeduloWithControls,ACC,ra,-XY,AVG,+300,A+B,FLN,-XY/GenomeWideSNP_6
Platform: Affymetrix
Chip type: GenomeWideSNP_6,Full,monocell
Number of arrays: 66
Names: control10, control11, ..., N813
Time period: 2008-11-06 20:37:47 -- 2008-11-06 20:37:56
Total file size: 1778.65MB
RAM: 0.11MB
Parameters: (probeModel: chr "pm", mergeStrands: logi TRUE, combineAlleles: logi TRUE)
Reference file:
<average across arrays>
RAM: 0.00MB
```

or using a paired reference set:

```
> gada <- GadaModel(ces1,cesReference,aAlpha=0.8,T=6,MinSegLen=3); #With reference
> print(gada)
```

the parameters aAlpha, T, and MinSegLen control the settings of the SBL and BackwardElimination methods as we described in this manual. To fit the model, we enter

```
> fit(gada,arrays=c(1,3,5),chromosomes=c(1,17,22),verbose=verbose)
```

or for the entire set of samples and chromosomes:

```
> fit(gada,verbose=verbose)
```

The results of the segmentation can be displayed using the graphical reporting tools implemented in aroma.affymetrix package:

```
> ceGada<- ChromosomeExplorer(gada)
> print(ceGada)
ChromosomeExplorer:
Name: MeduloWithControls
Tags: ACC,ra,-XY,AVG,+300,A+B,FLN,-XY,a0.8
Number of arrays: 66
Path: reports/MeduloWithControls/ACC,ra,-XY,AVG,+300,A+B,FLN,-XY,a0.8/GenomeWideSNP_6/gada
RAM: 0.00MB
> process(ceGada, chromosomes=c(19, 22, 23), verbose=verbose)
> display(ceGada)
```

The Firefox 2.0 or newer is required to visualize this results (Figure 22).

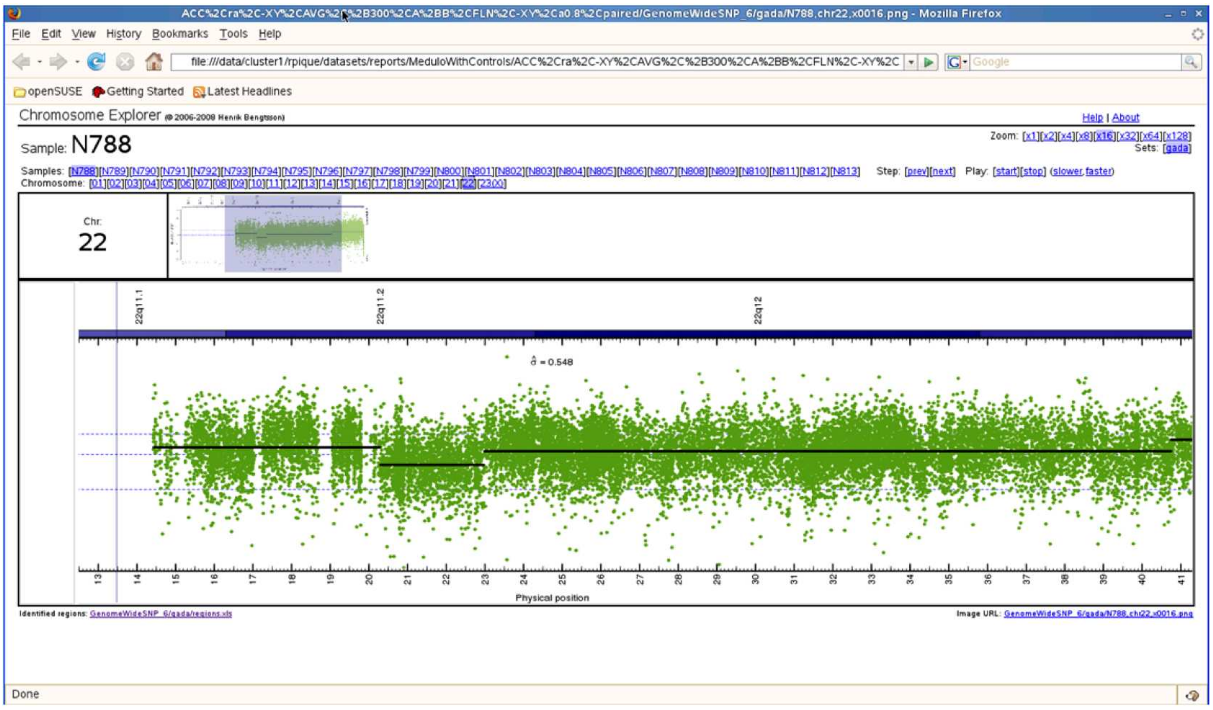

Figure 22: Browsing the segmentation results on aroma.affymetrix Chromosome Explorer

Alternatively the segments can be manually extracted to use in downstream analysis using:

```
> cnrs <- getRegions(gada, arrays=1, chromosomes=1, verbose=verbose)
```

```
 Extracting regions from all fits...
  Obtaining CN model fits (or fit if missing)...
  Obtaining CN model fits (or fit if missing)...done
  Extracting regions for chromosome #1...
  Extracting regions for chromosome #1...done
  Extracted regions:
  'data.frame': 22 obs. of  5 variables:
   $ chromosome: int  1 1 1 1 1 1 1 1 1 1 ...
   $ start     : num     51599 62900669 62923765 72541525 72570001 ...
   $ stop      : num  62900162 62922795 72541505 72569615 72582431 ...
   $ mean      : num  -0.0434 -1.1810 -0.0314 -2.4720 -1.7178 ...
   $ count     : num  37489     5  6739    26    15 ...
 Extracting regions from all fits...done
> print(cnrs)
$control10
   chromosome     start      stop        mean count\begin{figure}[h]
\begin{center}\label{fig-log-intensities}
\includegraphics[width=3in, height=\linewidth, angle=-90]{./graficas/log_intensities.eps}
\caption{Illumina log2ratio intensities by chromosome}
\end{center}
\end{figure}

1           1     51599  62900162 -0.04341482 37489
2           1  62900669  62922795 -1.18099960     5
3           1  62923765  72541505 -0.03139820  6739

...

21          1 241190561 241195976 -1.29550644     4
22          1 241201331 247191012 -0.04825901  3903
```

Note that GADA two step approach is, however, not fully exploited within the `aroma.affymetrix` framework. If we want to adjust `T` and `MinSegLen` to a higher value, we will obtain sparser results and reduce the FDR:

```
> cnrs <- getRegions(gada, arrays=1, chromosomes=1,T=20,MinSegLen=30)
 Repeating BackwardElimination()...
List of 2
 $ T        : num 20
 $ MinSegLen: num 30


> print(cnrs)
$control10
  chromosome    start      stop        mean  count
1          1    51599  72541505 -0.04171265  44233
2          1 72541525  72583737 -2.30345358     45
3          1 72584492 247191012 -0.04046404 102246

1         http://genome.ucsc.edu/cgi-bin/hgTracks?clade=vertebrate&org=Human&db=hg18&position=chr1
2         http://genome.ucsc.edu/cgi-bin/hgTracks?clade=vertebrate&org=Human&db=hg18&position=chr1
3         http://genome.ucsc.edu/cgi-bin/hgTracks?clade=vertebrate&org=Human&db=hg18&position=chr1
```

However, if we want to visualize the results with a higher value of T, we have to repeat the entire procedure.

```
> gada <- GadaModel(cesN,aAlpha=0.8,T=20,MinSegLen=30);
> process(ceGada, chromosomes=c(19, 22, 23), force=TRUE, verbose=verbose);
```

In a future versions will consider reusing the previously fitted SBL model to only repeat the backward elimination step.

References

[1] H. Bengtsson, R. Irizarry, B. Carvalho, and T. P. Speed. Estimation and assessment of raw copy numbers at the single locus level. *Bioinformatics*, 24(6):759–767, 2008.

[2] R. Pique-Regi, J. Monso-Varona, A. Ortega, R. C. Seeger, T. J. Triche, and S. Asgharzadeh. Sparse representation and bayesian detection of genome copy number alterations from microarray data. *Bioinformatics*, 24(3):309–18, 2008.

```
> toLatex(sessionInfo())
```

- R version 2.7.0 (2008-04-22), `i686-pc-linux-gnu`
- Locale: `LC_CTYPE=es_ES.UTF-8;LC_NUMERIC=C;LC_TIME=es_ES.UTF-8;LC_COLLATE=es_ES.UTF-8;LC_MONETARY=C;LC_[illegible]`
- Base packages: base, datasets, graphics, grDevices, methods, stats, utils
- Other packages: gada 0.7-4
- Loaded via a namespace (and not attached): tools 2.7.0
